# Active protection of a superconducting qubit with an interferometric Josephson isolator

**DOI:** 10.1038/s41467-019-11101-3

**Published:** 2019-07-17

**Authors:** Baleegh Abdo, Nicholas T. Bronn, Oblesh Jinka, Salvatore Olivadese, Antonio D. Córcoles, Vivekananda P. Adiga, Markus Brink, Russell E. Lake, Xian Wu, David P. Pappas, Jerry M. Chow

**Affiliations:** 1 IBM T. J. Watson Research Center, Yorktown Heights, New York, NY 10598 USA; 2 National Institute of Standards and Technology, Boulder, CO 80305 USA; 3 Present Address: Bluefors Oy, Arinatie 10, 00370 Helsinki, Finland

**Keywords:** Superconducting devices, Qubits

## Abstract

Nonreciprocal microwave devices play critical roles in high-fidelity, quantum-nondemolition (QND) measurement schemes. They impose unidirectional routing of readout signals and protect the quantum systems from unwanted noise originated by the output chain. However, cryogenic circulators and isolators are disadvantageous in scalable superconducting architectures because they use magnetic materials and strong magnetic fields. Here, we realize an active isolator formed by coupling two nondegenerate Josephson mixers in an interferometric scheme and driving them with phase-shifted, same-frequency pumps. By incorporating our Josephson-based isolator into a superconducting qubit setup, we demonstrate fast, high-fidelity, QND measurements of the qubit while providing 20 dB of protection within a bandwidth of 10 MHz against amplified noise reflected off the Josephson amplifier in the output chain. A moderate reduction of 35% is observed in *T*_2E_ when the Josephson-based isolator is turned on. Such a moderate degradation can be mitigated by minimizing heat dissipation in the pump lines.

## Abstract (web summary)

Magnetic-based isolators are critical components for protecting qubits against noise in quantum setups but unsuitable for large processors. Here, Abdo et al. show good protection of a qubit in a high-fidelity quantum readout setup using a Josephson-based isolator devoid of magnetic materials.

## Introduction

The capability to perform fast, high-fidelity, single-shot, quantum-nondemolition (QND) measurement of qubits is a critical requirement for the operation of quantum computers^1^. It is needed, for instance, to readout qubits in real-time^2,3^; track the evolution of quantum states^4,5^; detect error syndromes^6^; stabilize quantum states^7,8^; and apply quantum feedback, as required in certain protocols^9–11^. In the case of superconducting quantum processors, one prominent platform for performing QND measurements is circuit quantum electrodynamics (cQED)^12^, in which superconducting qubits are dispersively coupled to superconducting microwave readout resonators, and the qubit state is inferred by measuring the phase shift experienced by a weak near-resonance microwave signal applied to the readout resonator^13^. To perform such fast QND measurements in the cQED architecture, several key microwave components are commonly employed^14^, such as: (1) quantum-limited Josephson amplifiers, which enhance the signal to noise ratio of the output chain while adding minimal amount of noise to the processed signals^15,16^; (2) Purcell filters, which enable qubits to be coupled to fast readout resonators while inhibiting spontaneous emission of their excitations through the resonators^17,18^; and (3) nonreciprocal devices, which separate input from output and protect qubits against noise coming from the output chain^2,3^. However, unlike Josephson amplifiers or Purcell filters, which are compatible with superconducting circuits, have little or no internal loss, and can be miniaturized, nonreciprocal devices, i.e., cryogenic circulators and isolators, which are widely used in state-of-the-art quantum circuits, lack these desired properties. This is primarily because they rely on magneto-optical effects to break the transmission-coefficient symmetry for light under exchanging sources and detectors, which entail using magnetic materials and strong magnetic fields^19,20^.

In response to the immense challenge of eliminating these magnetic-based nonreciprocal devices, which severely hinder the scalability of superconducting quantum processors, a wide variety of viable alternative nonreciprocal schemes have been proposed and realized recently^21–42^. Examples of these schemes include: Josephson traveling-wave parametric amplifiers^24,25^; kinetic-inductance traveling-wave parametric amplifiers^26^; reconfigurable directional amplifiers and circulators based on three-mode Josephson devices^27–29^; Hall-effect-based circulators^30–32^; interferometric Josephson directional amplifiers^33–35^; circulators and directional amplifiers that are based on nanomechanical systems^36–38^; Josephson-array transmission-line isolator^39^; SQUID-based directional amplifiers^40^; or SQUID-variant devices, such as Superconducting Low-inductance Undulatory Galvanometer (SLUG) amplifiers^41^; and circulators, which rely on variable inductors^42^.

Here, we introduce a Josephson-based isolator, which is devoid of magnetic materials and strong magnets. The device is fully compatible with superconducting circuits and can be integrated on chip. It breaks reciprocity by generating artificial gauge-invariant potential for microwave photons by parametrically modulating the inductive coupling between two modes of the system and achieves unidirectional transmission of propagating signals by creating constructive and destructive wave interference between different paths in the device. The isolator has two key differences, compared to other Josephson-based, microwave-controlled circulators realized recently^27,29^, it preserves the frequency of the routed quantum signals and can be operated by a single monochromatic microwave drive instead of three. These differences can result in a significant reduction in the overall control hardware resource for operating a larger number of devices. Furthermore, here we test our Josephson-based isolator in a quantum setup. More specifically, we couple it to a superconducting qubit in a fast, high-fidelity measurement setup and demonstrate that, indeed, it provides active protection to the qubit against unwanted noise coming from the output chain.

## Results

### Nonreciprocity mechanism

**Fig. 1:**
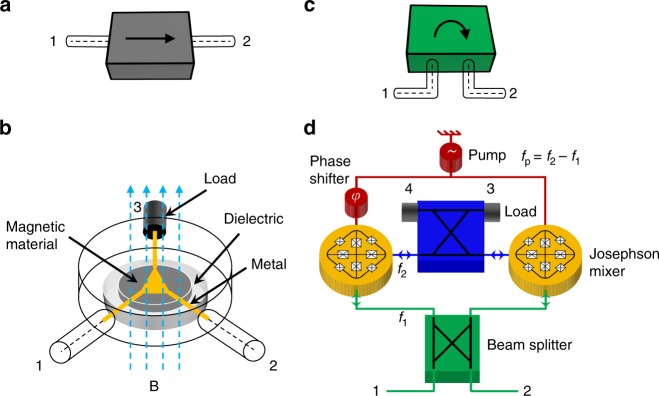
Magnetic-based isolator versus the MPIJIS. A microwave isolator, whose circuit symbol is shown in **a**, is a unidirectional, two-port microwave device. It transmits signals from port 1 to 2 (as indicated by the arrow) and blocks signals propagating in the opposite direction. To break the transmission-coefficient symmetry between source (input) and detector (output), commercial isolators rely on magneto-optical effects, which require magnetic materials and strong magnets. One common realization of such isolators is a three-port circulator in which one of its ports (i.e., 3) is terminated by a 50 Ω load as shown in **b**. In this example, signals entering port 1 are directed towards port 2, whereas signals entering port 2 are dissipated in the matched load attached to the internal port 3. **c** A circuit symbol for the Josephson-based isolator realized and measured in this work, which is based on a different reciprocity-breaking mechanism. In this device, schematically shown in **d**, two active Josephson mixers are coupled via beam-splitters and driven by a microwave pump source. Unidirectional transmission from port 1 to 2 is generated by constructive and destructive wave interference between different paths in the device. The interference pattern is controlled by the phase difference *φ* = −*π*/2 between the pumps feeding the two mixing stages. Signals propagating from port 2 to 1 are dissipated in the matched loads connected to the internal ports 3 and 4

To elucidate the basic idea behind the reciprocity-breaking mechanism of our isolator, we qualitatively compare in Fig. 1 a state-of-the-art magnetic-based isolator, whose circuit symbol is shown in Fig. 1a and the Multi-Path Interferometric Josephson ISolator (MPIJIS) realized and measured in this work, whose circuit symbol is introduced in Fig. 1c. While the circuit of the magnetic-based isolator can be realized in several different ways^20^, Fig. 1b exhibits a widely-used commercial realization, which captures the main common attributes of the device. In this realization, a two-port isolator is formed by terminating one port (e.g., port 3) of a three-port, magnetic-based circulator with a matched load (e.g., 50 Ω). As seen in Fig. 1b, the circulator consists of a microstrip metallic junction connected to the center-conductor of the equally-spaced device ports and laid down on a disk-shaped dielectric substrate, which incorporates, at its center, a smaller ferrite disk functioning as a resonant cavity. The circulator circuit also includes a permanent magnet, which induces a magnetic field in the device. As a result of the magnetic bias, the lowest-order resonant mode *f*_0_ of the ferrite cavity splits into two modes having different frequencies *f*_±_ and nonreciprocal azimuthal phase dependence *e*^±*jϕ*^, which is set by the direction of the applied magnetic field. By engineering the amplitude of these modes, via the bias field, a superposition pattern can be generated at the circulator ports, such that microwave signals at *f*_1_, falling between the split-resonance frequencies *f*_−_ < *f*_1_ < *f*_+_, propagate towards port 2 upon entering port 1, whereas signals entering 2 propagate towards the internal port 3 and dissipate their energy at the matched load^20^.

The MPIJIS relies, in contrast, on different physics. In Fig. 1d, we depict the main components of the device. Unlike the passive magnetic-based isolator shown in Fig. 1b, the MPIJIS consists of two active Josephson mixers coupled together in an interferometric setup formed by two beam-splitters (i.e., 90° hybrids). The two Josephson mixers function as lossless frequency converters (without photon gain) between two signals having frequencies *f*_1_ (green) and *f*_2_ (blue), which are supported by the resonance modes of the mixers. By construction, signals at *f*_2_ only excite an internal mode of the system. To enable the frequency conversion, the Josephson mixers are driven by a monochromatic microwave pump at frequency *f*_p_ = *f*_2_ − *f*_1_. Due to the parametric modulation of the Josephson mixers, an artificial gauge-invariant potential for microwave photons is generated, which depends on the pump-phase difference *φ*^27–29,43,44^. The induced potential consequently introduces a nonreciprocal phase shift for the frequency-converted signals^45^, which is exploited in combination with wave interference (enabled by the beam-splitters) for transmitting signals in one direction, i.e., from port 1 to 2, and canceling signals in the opposite direction. Similar to the magnetic-based isolator (Fig. 1b), the canceled signals on the device port 1 are directed towards the MPIJIS internal ports 3 and 4 where they are absorbed by the matched loads.

### The device

**Fig. 2:**
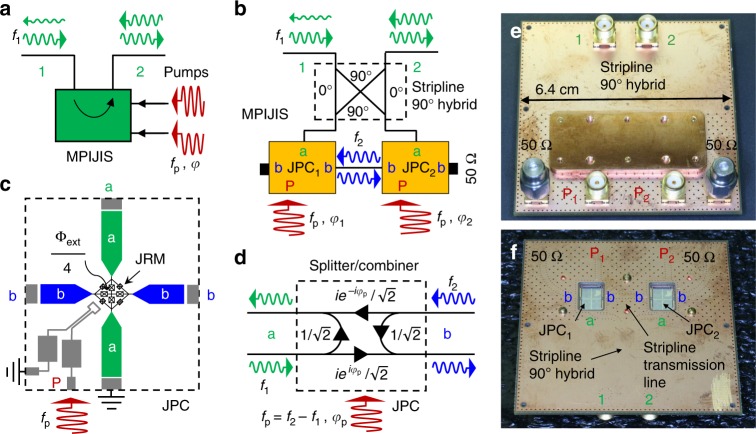
The MPIJIS device. **a** Black-box illustration of the MPIJIS operation. The MPIJIS receives two microwave drives (pumps) at the same-frequency *f*_p_ having a certain balanced amplitude and phase difference *φ* = −*π*/2. The MPIJIS transmits signals within the device bandwidth, e.g., *f*_1_, from port 1 to 2 with almost unity transmission, while significantly attenuating signals propagating in the opposite direction. **b** Block-circuit diagram of the MPIJIS measured in this work. The device consists of two Josephson parametric converters (JPCs), whose ports ‘a’ and ‘b’ are coupled by a 90° hybrid and a short transmission line, respectively, forming an interferometric setup for propagating signals at frequencies *f*_1_ and *f*_2_. One side of port ‘b’ of each JPC is terminated with a matched 50 Ω cold load forming the internal ports of the device. The pump is fed to each JPC through a separate physical port. **c** Schematic layout of the JPC employed in the MPIJIS. The JPC consists of two half-wavelength microstrip resonators, denoted as ‘a’ and ‘b’, strongly coupled to a JRM. Each resonator is capacitively coupled to one external feedline on either side via gap capacitances. Resonator ‘a’ is made single ended by shorting one of its feedlines to ground. The pump is injected to the JRM through an on-chip drive line. **d** Signal flow graph for the JPC operated at a 50:50 beam splitter/combiner working point, in which half of the input signal, on each port, is reflected off and the other half is transmitted to the other port with frequency conversion. The transmitted signal acquires a nonreciprocal phase shift based on the pump phase. **e**, **f** exhibit photos of the top and bottom view of the MPIJIS, respectively, realized by embedding two JPC chips into a multilayer printed circuit board, which incorporates a stripline 90° hybrid and transmission lines (the center-conductor of the stripline components is buried and therefore not visible). In **f**, the bottom cover that encloses the chips is removed to expose the JPCs

The MPIJIS realized in this work is shown in Fig. 2. In Fig. 2a, we show a black-box representation of the MPIJIS. It includes two external ports 1 and 2, which support propagating microwave signals at *f*_1_, which fall within the device bandwidth. The MPIJIS receives, for its operation, two monochromatic microwave pumps at *f*_p_ having a certain amplitude and phase difference *φ* = ±*π*/2. The pumps are fed into the MPIJIS through auxiliary ports. In the example of Fig. 2a, the MPIJIS is operated in the forward direction, i.e., propagating signals entering port 1 (input) are transmitted in the direction of the arrow to port 2 (output) with almost unity transmission, whereas propagating signals entering port 2 are significantly attenuated upon exiting port 1. Figure 2b exhibits a block-circuit diagram of the MPIJIS. It consists of two nominally identical Josephson parametric converters (JPCs)^46,47^, which serve as lossless, nondegenerate, three-wave mixing Josephson devices as outlined in Fig. 1d. The two JPCs are embedded into an interferometric setup, which couples modes *a* and *b* via a 90° hybrid and a short transmission line, respectively. The pump drives are fed to the JPCs via separate physical ports (denoted as P)^48^. The circuit diagram also reveals two internal ports of the device coupled to ports ‘b’ and terminated by 50 Ω loads. A schematic layout of the JPC is shown in Fig. 2c. It consists of two half-wavelength, microstrip resonators ‘a’ and ‘b’, which intersect at the center at a Josephson ring modulator (JRM)^49^. The JRM consists of four outer-loop Josephson junctions arranged in a Wheatstone bridge configuration. The four internal Josephson junctions inductively shunt the JRM and enable the resonance frequencies *ω*_a,b_/2*π* of resonators ‘a’ and ‘b’ to be tuned as a function of the applied external magnetic flux Φ_ext_ threading the JRM^50^. When 0 < |Φ_ext_| < Φ_0_, where Φ_0_ is the flux quantum, the JRM acts as a dispersive nonlinear medium mixing three orthogonal modes with a leading nonlinear term in the system Hamiltonian of the form ${\cal{H}}_{{\mathrm{3wave}}} = \hbar g_{\mathrm{3}}(a + a^\dagger )(b + b^\dagger)(c + c^\dagger )$^46^. Here, *g*_3_ is a flux-dependent coupling strength, *a* and *b* are the annihilation operators for the differential modes *a* and *b*, while *c* is the annihilation operator for the mode *c* common to both resonators. Each resonator is capacitively coupled, via equal gap capacitors, to two 50 Ω feedlines, which carry incoming and outgoing signals. The photon decay rates $\kappa _{{\mathrm{a,b}}}/2\pi \simeq 40\,{\mathrm{MHz}}$ of resonators ‘a’ and ‘b’ are primarily determined by the impedance mismatch created by the gap capacitors between the feedlines and resonators. In the MPIJIS configuration shown in Fig. 2b, resonator ‘a’ is single ended (i.e., one feedline is shorted to ground) and the pump drive is directly injected to the JRM through a separate on-chip feedline as illustrated in Fig. 2c^48^. When the JPC is operated as a frequency converter between modes *a* and *b*, a strong, coherent, off-resonant, common drive is applied at *ω*_c_ ≡ *ω*_p_ = *ω*_b_ − *ω*_a_^51,52^. With such a classical drive, we obtain in the rotating wave approximation ${\cal{H}}_{{\mathrm{3wave}}} = \hbar |g_{{\mathrm{ab}}}|(e^{i\varphi _{\mathrm{p}}}ab^\dagger + e^{ - i\varphi _{\mathrm{p}}}a^\dagger b)$, where *g*_ab_ is a pump-amplitude-dependent coupling strength and *φ*_p_ is the pump phase. On resonance, the transmission amplitude associated with this frequency-conversion process is given by *t* = 2*ρ*/(1 + *ρ*^2^), where $\rho = |g_{{\mathrm{ab}}}|/\sqrt {\kappa _{\mathrm{a}}\kappa _{\mathrm{b}}}$. The transmission amplitude varies between 0 (no conversion) and 1 (full conversion). Since this process is unitary, the reflection and transmission amplitudes satisfy the condition |*r*|^2^ + |*t*|^2^ = 1. Another crucial property exhibited by ${\cal{H}}_{{\mathrm{3wave}}}$ is the pump-phase-dependent, nonreciprocal phase shift imprinted on signals undergoing upconversion from mode *a* to *b* (*φ*_p_) versus downconversion from *b* to *a* (−*φ*_p_). This nonreciprocal phase shift is outlined in Fig. 2d, which shows a signal flow graph for the JPC operated at a special frequency-conversion working point known as the 50:50 beam splitter point, where $r = t = 1/\sqrt 2$.

By coupling the two JPCs in an interferometric setup as shown in the block-circuit diagram depicted in Fig. 2b (or device photos exhibited in Fig. 2e, f), we convert the nonreciprocal phase shift acquired by signals transversing the two JPCs, ±*φ*, where *φ* ≡ *φ*_1_ − *φ*_2_ is the phase gradient between the two pumps feeding the two mixers^45^, into a nonreciprocal amplitude response via constructive or destructive wave interference between multiple paths in the device. When solving the signal flow graph for the whole device (see Supplementary Note 1 and Supplementary Figs. 1 and 2), we get, on resonance, the following transmission parameters for ports 1 and 2,

1 $$S_{2 \rightleftarrows 1} = i\frac{{\sqrt {1 - t^2} \mp \sqrt 2 t^2\,{\mathrm{sin}}\, \varphi }}{{1 + t^2}},$$

and reflection parameters,

2 $$S_{11} = S_{22} = - i\frac{{\sqrt 2 t^2{\mathrm{cos}}\, \varphi }}{{1 + t^2}}.$$

In the special case in which the JPCs are operated at the 50:50 beam splitter point, i.e., $t = 1/\sqrt 2$, and the applied pump-phase difference is *φ* = −*π*/2, we obtain, by substituting into Eqs. (1), (2), total isolation of signals transmitted from port 2 to 1, *S*_12_ = 0, and vanishing reflections off ports 1 and 2, *S*_11_ = *S*_22_ = 0. Whereas, for signals transmitted from port 1 to 2, we obtain almost unity transmission $|S_{21}| = 2\sqrt 2 /3 \simeq 0.94$, which is equivalent to about 0.5 dB loss in signal power. A detailed calculation of the scattering matrix of the MPIJIS as well as key measurement results, i.e., Supplementary Notes 1, 2 and Supplementary Figs. 3 and 4.

It is worth noting that the same isolator circuit presented in Fig. 2b can be used to realize a quantum-limited, phase-preserving Josephson directional amplifier, which has recently been demonstrated in refs. ^33,34^. One main difference between the two interferometric nonreciprocal devices, relates to the mode of operation of the coupled JPCs. Here, the JPCs are operated in the frequency-conversion mode with no photon gain^46,51^, whereas in the directional amplifier application, they are operated in the nondegenerate amplification mode, where *ω*_p_ = *ω*_a_ + *ω*_b_^46,47,49^. As a direct consequence of this difference, the two nonreciprocal devices differ in two important aspects, namely, added noise and stability. While the phase-preserving directional amplifier is required by quantum mechanics to add noise equivalent to a half input photon at the signal frequency *n*_add_ = 1/2, the added noise by the isolator is mainly set by its power attenuation in the forward direction |*S*_21_|^2^ ^15,16,51^. In the MPIJIS case, the added noise-equivalent-input-photons at the signal frequency is given by *n*_add_ = (1 − |*S*_21_|^2^)/2|*S*_21_|^2^ (see Supplementary Note 3). For an ideal MPIJIS, whose JPCs are joined by symmetric couplers and operated at the 50:50 beam splitter point, we obtain *n*_add_ = 1/16 (corresponding to |*S*_21_|^2^ = 8/9 on resonance). Moreover, in the directional amplifier case, the amplitude-gain of each JPC is bounded by the amplitude-attenuation of the internal mode *b*. This is to ensure the device stability in the presence of the feedback loop formed between the JPCs^33,34^. Such a stability requirement is not applicable in the isolation case.

It is also worth noting that the dual operation of the present device, as a directional amplifier or an isolator (depending on the pump frequency, amplitude, and phase), is similar to other nonreciprocal Josephson devices reported recently^27,29^.

### The quantum setup

**Fig. 3:**
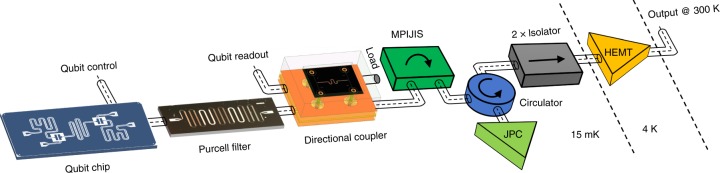
Rapid, high-fidelity, QND measurement setup incorporating the MPIJIS device. The setup, exhibiting only the main components, includes a two-qubit chip in which only one qubit is measured. The measured qubit is read through a single-port, fast readout resonator and controlled via a separate line, which is capacitively coupled to the qubit. To prevent the qubit from relaxing through the readout resonator, a Purcell filter is added to the resonator port, which connects to the rest of the setup. To enable a reflective measurement of the qubit without utilizing a circulator, a broadband, on-chip directional coupler is incorporated between the Purcell filter and the readout input line. In this configuration, the readout signals input on the directional coupler couple, with about 20 dB of attenuation, to the Purcell filter (and readout resonator), whereas the output readout signals reflecting off the readout resonator are transmitted, with a small insertion loss, to a MPIJIS incorporated into the output chain. Following the MPIJIS, a cryogenic circulator is used to route the readout signal to a quantum-limited Josephson amplifier working in reflection (i.e., JPC) and route the amplified reflected signal off the JPC towards the following amplification stage along the output chain, i.e., the HEMT, which is separated from the JPC circulator by two wideband cryogenic isolators, which further protect the qubit from noise coming down the output chain

To demonstrate that the MPIJIS is suitable for quantum applications, we incorporate it into a high-fidelity qubit measurement setup as shown in Fig. 3. The qubit chip used in the measurement consists of two coupled transmons, but only one transmon is measured. Each qubit has its own capacitively coupled control port, which is separate from the readout resonator port used for measurement. To enable fast readout, the qubit is dispersively coupled to a relatively large bandwidth readout resonator which, in turn, is coupled to a superconducting Purcell filter integrated into the same PCB as the qubit chip (see the Methods section). The Purcell filter is added to preserve the qubit lifetime by suppressing spontaneous emission of qubit excitations through the fast resonator. The effective readout bandwidth of the combined resonator-Purcell system is *κ*/2*π* = 6.6 MHz. The qubit and readout frequencies are *ω*_q_/2*π* = 5.3788 GHz and *ω*_r_/2*π* = 6.8405 GHz, respectively, while the qubit-state-dependent resonance frequency shift is *χ*/2*π* = 4.2 MHz. Another important novelty of this qubit setup is the integration of a custom-made, wideband, superconducting directional coupler to couple readout signals into and out of the readout resonator. Compared to conventional qubit setups, which utilize a circulator to measure readout resonators in reflection, using a directional coupler produces two key differences: (1) the input readout signal entering the directional coupler is attenuated by about 18 dB, which can be effectively lumped into the total attenuation of the input readout line, and (2) the directional coupler is reciprocal, therefore, does not protect the qubit against noise coming from the output chain. The main advantages of using a directional coupler over a magnetic circulator are compatibility with superconducting quantum circuits and integrability with other microwave components in the measurement scheme. Further information about the directional coupler performance, fabrication, and packaging can be found in the Methods section. Following the directional coupler, which transmits the output readout signal with minimal attenuation, we incorporate the MPIJIS and a quantum-limited Josephson amplifier (i.e., JPC), separated by a cryogenic circulator. The circulator is crucial, in this case, for three reasons: (1) it separates the weak readout signal input on the JPC from the amplified reflected output signal; (2) it preserves, to a large extent, the output signal power due to its relatively low insertion loss (<1 dB); and (3) it partially protects the qubit (by about 15–18 dB) against vacuum noise amplified in reflection by the JPC.

### Qubit measurements

**Fig. 4:**
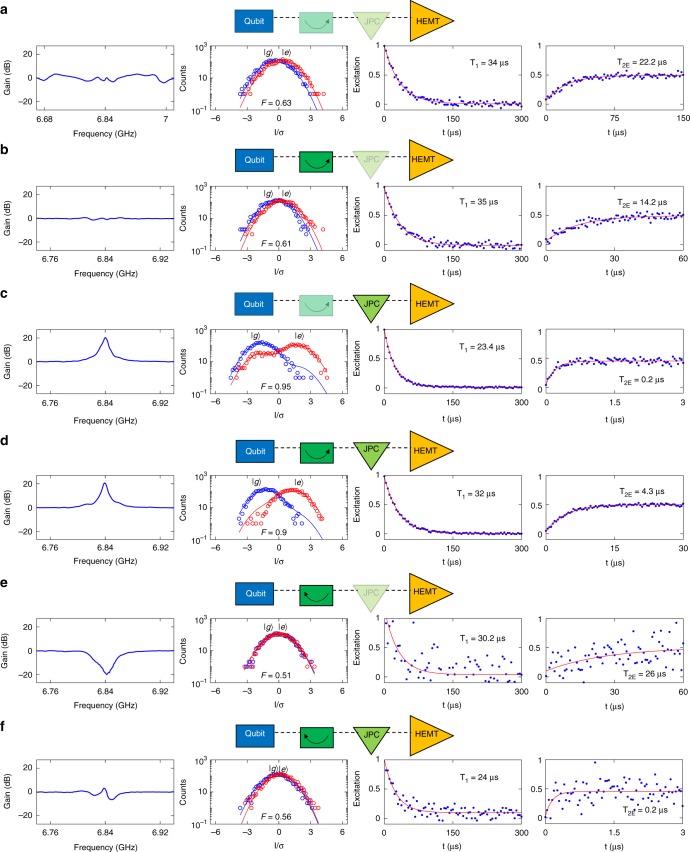
Qubit measurement with the MPIJIS. Panels **a**–**f** outline the six possible configurations of the qubit measurement setup shown in Fig. 3. In **a**, the MPIJIS and JPC are OFF. In **b**, the MPIJIS is ON and biased in the forward direction while the JPC is OFF. In **c**, the MPIJIS is OFF while the JPC is ON. In **d**, the MPIJIS and JPC are ON and the MPIJIS is biased in the forward direction. In **e**, the MPIJIS is ON and biased in the backward direction while the JPC is OFF. In **f**, the MPIJIS and JPC are ON and the MPIJIS is biased in the backward direction. The measurement results exhibited in the four columns for each configuration correspond to the net gain of the reflected readout signal relative to the HEMT-only case (first column from the left), the qubit readout fidelity measurement (second column), *T*_1_ (third column) and *T*_2E_ of the qubit (fourth column). The measured histograms, in the second column, for the initialized ground (|*g*〉) and excited (|*e*〉) states are depicted using blue and red circles, respectively. The blue and red solid curves represent Gaussian fits. The red solid curves in the third and fourth columns represent exponential fits to the blue data points

Figure 4 shows the main results of this work taken with the experimental setup of Fig. 3. Figure 4a–f outlines the six possible measurement configurations of the MPIJIS and the JPC. The graphs, displayed in the four columns, exhibit measurement results taken for each configuration, namely, normalized gain/attenuation of the MPIJIS and JPC versus frequency (first column from the left), readout fidelity histograms of the output chain extracted from the in-phase quadrature (I) of the output field corresponding to the qubit being initialized in the ground (|*g*〉) and excited (|*e*〉) states (second column), relaxation time *T*_1_ (third column), and decoherence time *T*_2_ echo of the qubit (fourth column). In configuration **a**, in which both the MPIJIS and JPC are OFF, we obtain an almost flat transmission parameter because the MPIJIS is transparent for propagating signals and the JPC is totally reflective. Using this configuration, we obtain baseline values for the readout fidelity 0.63, *T*_1_ = 34 μs and *T*_2E_ = 22.2 μs. Similar readout fidelities and coherence times are measured for this qubit in a separate cooldown when using the conventional high-fidelity setup instead, in which a cryogenic circulator and isolator replace the superconducting directional coupler and the MPIJIS, respectively (see Supplementary Note 4 and Supplementary Fig. 5). In configuration **b**, we operate the MPIJIS in the forward direction while keeping the JPC OFF. In this case, we observe a small dip in the transmission near resonance of about 0.7 dB, which is close to the ideal value of 0.5 dB. The observed small dip also explains the slight decrease in the readout fidelity to 0.61 (seen in the second column). While we do not observe a decrease in the qubit lifetime due to the operation of the MPIJIS as seen in the third column, we do observe about 35% reduction in *T*_2E_ = 14.2 μs, which we attribute to increased qubit dephasing as a result of heat dissipation in the pump lines of the MPIJIS (see Supplementary Note 5 and Supplementary Figs. 6 and 7). In configuration **c**, we turn off the MPIJIS and turn on the JPC with a gain of 20.7 dB at the readout frequency and a dynamical bandwidth of about 6 MHz, as shown in the first column. As expected in this quantum-limited amplification scenario, the readout fidelity is significantly enhanced *F* = 0.95. However, the enhancement in the fidelity is accompanied by a strong backaction on the qubit state, as seen in the drop of *T*_1_ = 23.4 μs and the significant reduction of *T*_2E_ = 0.2 μs by about a factor of 100, compared to configuration **a**. This backaction is caused by amplified in-band quantum noise, which couples to the resonator-qubit system due to insufficient isolation (i.e., only one isolation stage is present between the qubit and JPC). Next, in configuration **d**, we keep the JPC ON and operate the MPIJIS in the forward direction. Remarkably, by turning on the active isolation, *T*_1_ = 32 μs is almost restored to the baseline-value of configuration **a**, while *T*_2E_ = 4.3 μs is enhanced by a factor of 22 compared to configuration **c** (i.e., without the active isolation). Moreover, the observed enhancements in the coherence times of the qubit, compared to configuration **c**, are achieved without a considerable impact on the total gain 20 dB and readout fidelity *F* = 0.9. In the last two remaining configurations **e** and **f**, the MPIJIS is operated in the backward direction, which is attained in situ by shifting the pump-phase difference of the two drives by *π* compared to configurations **b** and **d**. In this mode of operation, the MPIJIS effectively mimics an attenuator of about 20 dB in the path of the readout signals as seen in the first column of configuration **e** for which the JPC is OFF. As expected in the case of heavy attenuation of configuration **e**, the readout fidelity is diminished *F* = 0.51. A similar effect can also be seen in the poor signal to noise ratio of the coherence times measurements, which have a large scatter in the data points (despite being averaged more than the other cases). In general, the coherence times measured for the backward-operated isolator *T*_1_ = 30.2 μs, *T*_2E_ = 26 μs are roughly comparable to those of the forward-operated isolator (i.e., configuration **b**). Finally, in configuration **f**, in which the JPC is ON, the amplification of the JPC almost cancels the attenuation of the MPIJIS as seen in the first column, which leads to a slight enhancement of the readout fidelity *F* = 0.56. The coherence times *T*_1_ = 24 μs, *T*_2E_ = 0.2 μs, measured for this case, are quite similar to configuration **c**. This is because in both cases the isolator is not shielding the qubit against excess backaction of the preamplifier (JPC).

To quantify the backaction effect on *T*_2E_ corresponding to the different configurations, we note that because 1/*T*_2E_ = 1/2*T*_1_ + 1/*T*_*ϕ*_ and *T*_2E_ < 2*T*_1_, it follows that the qubit coherence time in our case is limited by dephasing, where *T*_*ϕ*_ is the dephasing time. One dominant dephasing mechanism in a qubit-resonator system operating in the dispersive coupling regime, such as ours, is photon shot noise in the resonator mode, which causes the qubit frequency to fluctuate due to the AC Stark effect^53–56^. In this case, the dephasing rate Γ_*ϕ*_ ≡ 1/*T*_*ϕ*_ is given by $\Gamma _\phi = \bar n\kappa \chi ^2/(\kappa ^2 + \chi ^2)$, where $\bar n$ is the average photon number in the resonator, which can be written as $\bar n = \bar n_{{\mathrm{th}}} + \bar n_{{\mathrm{ba}}}$, where $\bar n_{{\mathrm{th}}}$ and $\bar n_{{\mathrm{ba}}}$ are the average thermal and nonthermal (backaction) photon numbers, respectively. Using these relations, we can evaluate $T_\varphi$, $\bar n_{{\mathrm{th}}}$, and $\bar n_{{\mathrm{ba}}}$, summarized in Table 1, for the different configurations **a**–**f** exhibited in Fig. 4. Note that in Table 1, we assume that excess dephasing is caused by either an increased thermal photon population in the readout resonator, due to generated heat in the pump lines feeding the MPIJIS (an experimental justification for this assumption is included in the Supplementary Information), and/or by amplified quantum noise reflecting off the JPC port and leaking into the readout resonator, as a result of insufficient isolation (backaction photons). As expected, the largest value for $\bar n_{{\mathrm{ba}}}$ = 0.41 is obtained for configurations **c** and **f** in which the JPC is ON while the MPIJIS is OFF or operated in the backward direction (i.e., in a nonprotective mode). Conversely, a significant reduction in the backaction photon number, by more than one order of magnitude 0.01, is observed when the MPIJIS is operated in the protective forward direction (i.e., configuration **d**).

**Table 1:** Coherence and backaction comparison

Configuration	MPIJIS	JPC	*T*_1_ (μs)	*T*_2E_ (μs)	*T*_*ϕ*_ (μs)	${\bar{\boldsymbol{n}}}_{{\bf{th}}}$	${\bar{\boldsymbol{n}}}_{{\bf{ba}}}$
**a**	OFF	OFF	34	22.2	32.9	0.003	0
**b**	F	OFF	35	14.2	17.8	0.005	0
**c**	OFF	ON	23.4	0.2	0.2	0.003	0.41
**d**	F	ON	32	4.3	4.6	0.005	0.01
**e**	B	OFF	30.2	26	45	0.002	0
**f**	B	ON	24	0.2	0.2	0.002	0.41

Evaluated *T*_*ϕ*_, $\bar n_{{\mathrm{th}}}$, and $\bar n_{{\mathrm{ba}}}$ for the different configurations. The symbols F and B correspond to the MPIJIS operated in the forward and backward direction, respectively

## Discussion

Based on the results of Fig. 4 and Supplementary Figs. 3, 6, and 7, there are several areas in which the performance of this proof-of-principle MPIJIS can be further improved: (1) the moderate reduction in *T*_2E_ (of about 35%) when the isolator is ON (Fig. 4b) versus OFF (Fig. 4a). Based on the measurement results of Supplementary Figs. 6 and 7, this reduction can be attributed to increased thermal population in the readout resonator due to heat generated in the pump lines feeding the MPIJIS. Such a heating effect can be mitigated, for example, by replacing the normal-metal directional couplers incorporated into the pump lines at the base-temperature stage (shown in Supplementary Fig. 6b) with superconducting ones and/or dissipating the pump power reflecting off the MPIJIS at a higher-temperature stage; (2) the excess insertion-loss observed for on-resonance transmitted signals at certain working points (as seen in Supplementary Fig. 3a,b). This excess loss, which is about 1 dB higher than the ideal-case scenario of 0.5 dB (see device theory in the Supplementary Information), suggests that the constructive interference taking place in the forward direction, for certain working points, is not optimal due to phase and amplitude imbalance of the PCB-hybrid^33^. By substituting the present unoptimized PCB-hybrid with an optimized on-chip version, we believe this figure and the reflections off the device ports (see Supplementary Fig. 3c, d) can be significantly reduced; and (3) the relatively narrow bandwidth of the MPIJIS, which is about 10 MHz and limited by the resonator bandwidths of the JPCs^46,51^. One method which could be employed to enhance the device bandwidth is impedance-engineering of the JPC feedlines. Applying such a technique in the case of single-port Josephson parametric amplifiers has successfully yielded bandwidths in the range 0.6–0.7 GHz^57,58^, which correspond to more than 12-fold enhancement compared to standard designs.

Additional enhancements of the device include, (1) reducing its footprint by integrating all components on chip and using lumped-element realization of the JPCs^59^ and hybrids^60^, (2) unifying the two external ports of the pumps, as shown in Fig. 2e,f. This could be achieved by injecting a single-pump drive into the MPIJIS through an on-chip 90° hybrid whose two output ports connect to the two-stage pump lines, as proposed in ref. ^45^.

In conclusion, we have introduced and realized a Josephson-based isolator device, which does not rely on magnetic materials or strong magnetic fields and is fully compatible with superconducting quantum circuits. The isolator is comprised of two coupled, nondegenerate, three-wave Josephson mixers embedded in an interferometric scheme. The nonreciprocal response of the device is controlled by the phase gradient of the same-frequency microwave drives feeding the two Josephson mixers. Such a microwave-signal control could enable fast switching of the isolation direction on the fly with a time scale on the order of 15 ns, which is mainly limited by the inverse dynamical bandwidth of the device. The realized isolator exhibits isolation in excess of 20 dB, a dynamical bandwidth of 10 MHz, insertion loss of about 0.7 dB in the forward direction, signal-power reflections off its input and output ports below −10 dB, and a maximum input power of −108 dBm (see Supplementary Fig. 4). Furthermore, we have validated the applicability of this isolation scheme for quantum measurements by incorporating it into a superconducting qubit measurement setup, which includes a transmon coupled to a fast cavity, Purcell filter, custom-made, broadband, superconducting directional coupler, and a quantum-limited, Josephson parametric amplifier. Using this novel setup, we have demonstrated fast, single-shot, high-fidelity, QND measurements of the quantum state while providing active protection of the qubit against amplified noise originated by the Josephson parametric amplifier. Owing to its numerous desired properties, an optimized version of this Josephson isolator may play a pivotal role in scalable quantum architectures.

## Methods

### Qubit measurement parameters

The frequencies of the pumps applied to the JPC (amplifier) and MPIJIS, in the measurements of Fig. 4, are $16.991$ GHz and 2.784 GHz, respectively. All qubit data exhibited in Fig. 4 is taken with a readout pulse duration of *t*_r_ = 200 ns, integration time of *t*_int_ = 200 ns, and an average photon number in the readout resonator of $\bar n = 2.1$. The qubit data are averaged over 2000 iterations. Using the expression ${\mathrm{tan}}\, (\theta /2) = \chi /\kappa$, we extract a qubit-state-dependent phase shift of the readout signal of *θ* = 64°. Furthermore, by substituting the histogram peak location ${\bar{\mathrm{I}}}/\sigma = 1.7$ for configuration **c** in Fig. 4 (in which the JPC is ON) in the relation ${\bar{\mathrm{I}}}/\sigma = \sqrt {2\bar n\eta \kappa t_{{\mathrm{int}}}} {\mathrm{sin}}\, (\theta /2)$^3^, we get an approximate value for the measurement efficiency of our readout chain $\eta \simeq 0.3$.

### Scattering parameters of the directional coupler

**Fig. 5:**
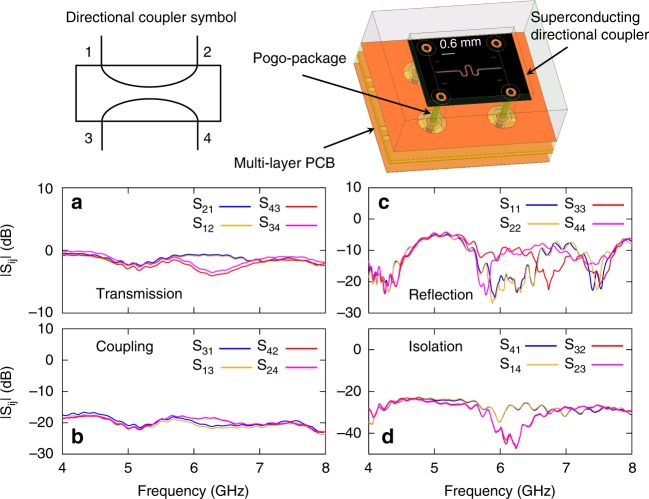
Characterization measurement of the on-chip, superconducting directional coupler. The scattering parameters of the device are measured in a separate cooldown using four pairs of calibrated input and output lines, where each pair connects to one of the device ports shown in the top-left corner of the figure, through a three-port, commercial circulator. In the top-right corner of the figure, we exhibit a schematic stack-up of the device, which consists of a superconducting directional coupler chip and multilayer PCB which carries the incoming and outgoing signals via SMA connectors. The device also consists of a pogo-package which encloses the chip and connects between the directional coupler ports and designated metallic traces within the multilayer PCB (see text and Fig. 6). **a** shows the measured transmission parameters of the directional coupler versus frequency |*S*_21_|^2^ (blue), |*S*_12_|^2^ (orange), |*S*_34_|^2^ (magenta), and |*S*_43_|^2^ (red). **b** shows the measured coupling parameters |*S*_31_|^2^ (blue), |*S*_13_|^2^ (orange), |*S*_24_|^2^ (magenta), and |*S*_42_|^2^ (red). **c** shows the measured reflection parameters |*S*_11_|^2^ (blue), |*S*_22_|^2^ (orange), |*S*_44_|^2^ (magenta), and |*S*_33_|^2^ (red). **c** shows the measured isolation parameters |*S*_41_|^2^ (blue), |*S*_14_|^2^ (orange), |*S*_23_|^2^ (magenta), and |*S*_32_|^2^ (red)

The superconducting, on-chip directional coupler is realized using coupled coplanar waveguides. The characterization results of the on-chip, wideband, superconducting directional coupler are shown in Fig. 5. A circuit symbol of the directional coupler is shown in the upper-left corner of the figure, which defines the four ports of the device. The characterization measurement is done in a separate cooldown without the qubit setup, where each port of the directional coupler is connected to a three-port, cryogenic circulator with its own input and output lines. A schematic image of the directional coupler chip mounted into a 50 Ω-matched pogo-package is shown in the upper-right corner. As seen in the image, the pogos (pins) inside the package connect the four ports of the directional coupler to designated copper traces in a multilayer PCB. The four copper traces, carrying incoming and outgoing signals, connect to surface-mount SMA connectors at the periphery of the PCB, which in our case define the directional coupler ports. In Fig. 5a–d, we exhibit calibrated measurements of the 16 scattering parameters of the directional coupler in the 4–8 GHz frequency range (limited by the bandwidth of cryogenic, three-port circulators employed in the measurement). The calibration of the transmission parameters is performed using a through measurement of the various pairs of lines used in the characterization of the directional coupler. The direct connection between the different pairs of lines is changed over three consecutive cooldowns. The calibration of the reflection parameters is performed in a fourth cooldown in which the lines used in the characterization are left open ended. Figure 5a exhibits the measured magnitude of the transmission parameters |*S*_21_|^2^ (blue), |*S*_12_|^2^ (orange), |*S*_34_|^2^ (magenta), and |*S*_43_|^2^ (red). The data show that the transmission through the device is close to 0 dB in a wide bandwidth, except for a narrow window around 6.3 GHz, where the minimum transmission between ports 3 and 4 is −4 dB. It is worth noting that this measurement includes the insertion loss of the multilayer PCB, whose dielectric material is FR4 (*ε*_*d*_ = 3.65). Figure 5b displays the measured coupling parameters |*S*_31_|^2^ (blue), |*S*_13_|^2^ (orange), |*S*_24_|^2^ (magenta), and |*S*_42_|^2^ (red). The data show that the coupling of the device is approximately flat across the bandwidth with an average value of about −19 dB. Figure 5c plots the measured reflection parameters |*S*_11_|^2^ (blue), |*S*_22_|^2^ (orange), |*S*_44_|^2^ (magenta), and |*S*_33_|^2^ (red). The data show that the reflection off the device ports varies across the measurement bandwidth in the −10 dB to −20 dB range. Finally, Fig. 5d depicts the isolation parameters of the device |*S*_41_|^2^ (blue), |*S*_14_|^2^ (orange), |*S*_23_|^2^ (magenta), and |*S*_32_|^2^ (red). As seen in the figure, the isolation varies between −25 to −40 dB in the measurement bandwidth, but averages around −30 dB.

### Directional coupler package

**Fig. 6:**
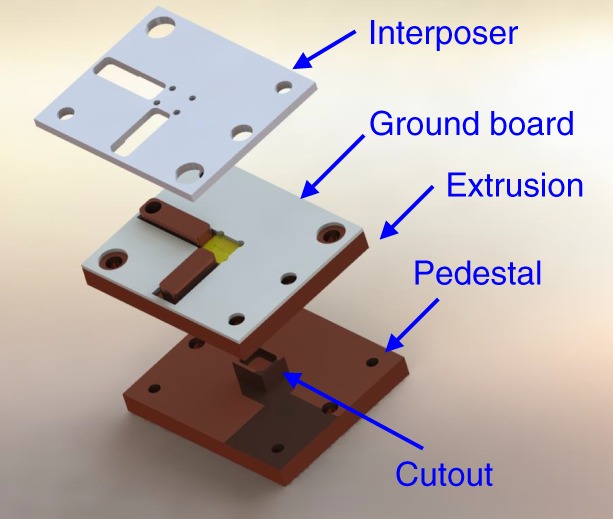
Illustration of the directional coupler package. It displays the different layers of the pogo-package used for housing the wideband, superconducting directional coupler. The pogo-package is used to provide 50 Ω matched vertical transitions between the directional coupler chip and its PCB

To preserve the wideband characteristics of the designed superconducting directional coupler when coupling its pads to 50 Ω PCB transmission lines, we utilize a pogo-package technology detailed in ref. ^61^, which provides good vertical 50 Ω impedance-matched transitions compared to traditional wirebond technology. A blow-up of the pogo-package used is shown in Fig. 6. It consists of, from bottom to top, a pedestal, an extrusion, a ground board, and an interposer. A cutout is included in the pedestal to push any chip modes to high frequency, and the chip is aligned to the two bosses on the extrusion. The ground board is made to press down on the edges for thermalization and has plating on the inside surfaces of the cutout to avoid exposed dielectric. The tolerances are adjusted so the chip protrudes slightly above the surface of the extrusion to establish positive contact with the ground board. Wirebonds are then added from the ground board to the chip ground plane and across the traces to suppress parasitic modes. The interposer is then clamped on top and spring-loaded pins with 50 Ω dielectrics are used for electrical connections^61^.

### The Purcell filter

The Purcell filter is realized using five sections of stepped impedance coplanar waveguide (CPW) transmission lines^18^. The CPW sections have alternating characteristic impedances and lengths, i.e., *Z*_lo_ = 25 Ω of length $\ell _{{\mathrm{lo}}} = 8.5\,{\mathrm{mm}}$, and *Z*_hi_ = 120 Ω of length $\ell _{{\mathrm{hi}}} = 6.5\,{\mathrm{mm}}$. The Purcell filter starts and ends with a low-impedance section. The device functions as a bandpass filter for readout frequencies spanning about 1 GHz of bandwidth around 6.5 GHz. Moreover, it suppresses signals in the frequency range 2–6 GHz. More specifically, it yields an attenuation of about 20 dB near the qubit frequency (~5.2 GHz)^18^. The Purcell filter is back-mounted into the same PCB as the qubit-resonator chip using a copper bottom cover. The qubit-resonator chip and the Purcell filter are coupled through a ~10 mm long 50 Ω stripline transmission line within the PCB.

### Fabrication process

The qubit-resonator circuit is fabricated on high-resistivity silicon by mixed optical and e-beam lithography. Large features of the superconducting quantum circuit are transferred into a thin film of niobium by optical lithography and reactive ion etch. Subsequently, the Al-Al_2_O_3_-Al Josephson junctions are defined by electron beam lithography and deposited by shadow mask evaporation in a Dolan-bridge process.

The directional coupler chip is fabricated with a single-layer optical lithography process using sputter-deposited Nb metal on a 3-inch, high-resistivity, float zone silicon substrate. Native oxide is removed from the substrate in situ with an Ar plasma immediately prior to deposition. The photoresist mask is then patterned on the surface of the Nb and the coplanar gaps are etched with SF_6_ plasma using laser-reflection endpoint detection. The photoresist mask is stripped in hot solvents. Finally, a protective coat of resist is applied before the wafer is diced into dies.

The Purcell filter is implemented using a SF_6_ subtractive dry etch of 200 nm thick niobium sputtered on a 4 × 10 mm^2^ sapphire substrate.

## Supplementary information

Supplementary Information

## Data Availability

The data that support the findings of this study are available from the corresponding author upon reasonable request.
