# Supplementary material for: Active protection of a superconducting qubit with an interferometric Josephson isolator
Source: Nat Commun. 2019 Jul 17;10:3154. doi: 10.1038/s41467-019-11101-3 (PMC6637130; doi:10.1038/s41467-019-11101-3)
Supplement: Supplementary file 1 — Supplementary Information [file 41467_2019_11101_MOESM1_ESM.pdf]

Supplementary Information for "Active protection of a superconducting qubit with an interferometric Josephson isolator"

Abdo et al.

Supplementary Note 1: MPIJIS theory

To calculate the scattering parameters of the MPIJIS and demonstrate its isolation operation, we use the effective signal-flow graph exhibited in Supplementary Figure 1a. The graph includes signal-flow graphs for the two coupled JPCs operated in frequency-conversion mode. On-resonance signals at $f_1 = f_\mathrm{a}$ or $f_2 = f_\mathrm{b}$ input on port a (e.g., $1'$ or $2'$) or b (e.g., b1 or b2) are reflected off by a reflection-parameter r and transmitted with frequency-conversion by a transmission-parameter t, where r and t are determined by the pump drive amplitude and satisfy the energy conservation condition $r^2 + t^2 = 1$. In this calculation, we assume that the two JPCs are balanced, i.e., their reflection and transmission parameters are equal. It is worth noting that although we consider the on-resonance case here, it is straightforward to generalize the device response for signals that lie within the JPC dynamical bandwidth as we show below. The nonreciprocal phases φ_1 and φ_2 acquired by the frequency-converted transmitted signals between ports a and b, as indicated in the graph, correspond to the phases of the pump drives at frequency f_p feeding JPC_1 and JPC_2, respectively. Supplementary Figure 1a also includes flow-graphs for two couplers coupling the a and b ports of the JPCs; one represents the 90° hybrid, which couples between the a ports of the JPCs, while the other is a fictitious one coupling the b ports. The main role of the latter coupler is to model the amplitude attenuation present on the b port α, due to signal absorption in the 50 Ω cold loads and the insertion loss of the normal-metal transmission line coupling the two stages. Because of the structural symmetry of our device, we consider a symmetric coupler with real coefficients α and β, which satisfy the condition $\alpha^2 + \beta^2 = 1$. For an ideal symmetric coupler (i.e., 90° hybrid), $\alpha = \beta = 1/\sqrt{2}$ [1].

In the stiff pump approximation, the JPC reflection and transmission parameter amplitudes can be written as [2]

$$\begin{aligned} r &= \frac{1-\rho^2}{1+\rho^2}, \\ t &= \frac{2\rho}{1+\rho^2}, \end{aligned} \tag{1}$$

where $0 \leq \rho \leq 1$ is a dimensionless pump amplitude. The lower bound $\rho = 0$ corresponds to the case of no applied pump in which the JPC is OFF and acts as a perfect mirror, whereas the upper bound $\rho = 1$ corresponds to the case of full frequency conversion mode between ports a and b. By inspection [1], the scattering matrix of the inner device defined by the ports $1', 2', 3, 4$, i.e., excluding the first 90° hybrid, can be written in the form [3]

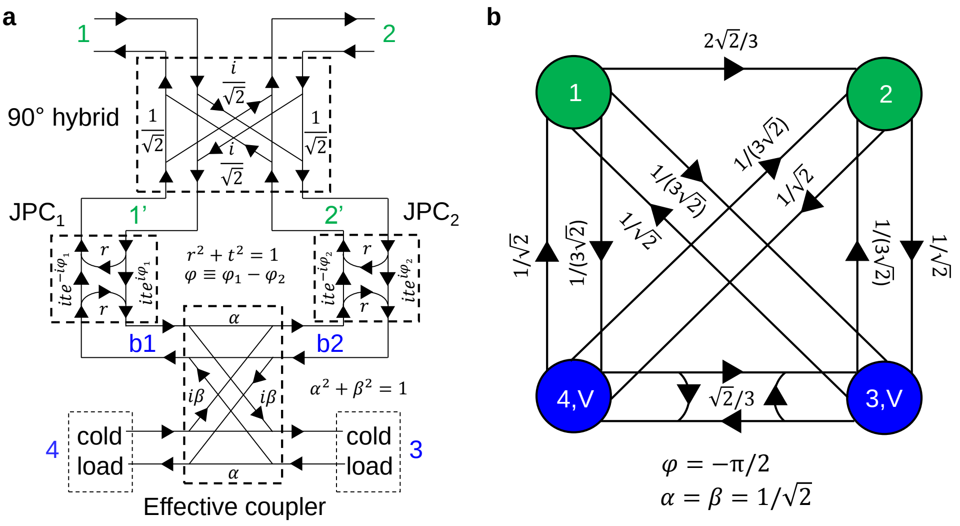

Supplementary Figure 1: **Graphical representations of the MPIJIS scattering parameters. a** Signal flow graph for the MPIJIS. It exhibits two JPCs operated in frequency conversion mode on resonance, i.e., $f_1 = f_{\mathrm{a}}$, $f_2 = f_{\mathrm{b}}$. Ports a of the JPCs, denoted as $1'$, $2'$, are coupled via a 90° hybrid while their internal b ports, denoted as b1 and b2, are coupled via a fictitious coupler, which models the attenuation present on the internal b channel of the MPIJIS due to dissipation in the 50 Ω cold terminations and the transmission line coupling the two JPCs. The coupler coefficients α and β are taken to be real, satisfying the condition $\alpha^2 + \beta^2 = 1$. The transmitted signals between ports a and b of the JPC undergo frequency conversion and acquire a nonreciprocal phase shift, which depends on the phase of the drive. **b** A graphical representation of the scattering parameters of the device for the case $r = t = 1/\sqrt{2}$ (the JPCs are operated at the 50:50 beam splitter working point), $\varphi = -\pi/2$, and the effective coupler is symmetrical and balanced, i.e., $\alpha = \beta = 1/\sqrt{2}$.

$$[s] = \begin{pmatrix} s_{1'1'} & s_{1'2'} & s_{1'3} & s_{1'4} \\ s_{2'1'} & s_{2'2'} & s_{2'3} & s_{2'4} \\ s_{31'} & s_{32'} & s_{33} & s_{34} \\ s_{41'} & s_{42'} & s_{43} & s_{44} \end{pmatrix}$$

$$= \begin{pmatrix} \frac{r\beta^2}{1-\alpha^2r^2} & -\frac{\alpha t^2 e^{-i\varphi}}{1-\alpha^2r^2} & -\frac{\beta t e^{-i\varphi_1}}{1-\alpha^2r^2} & -\frac{\beta r\alpha t e^{-i\varphi_1}}{1-\alpha^2r^2} \\ -\frac{\alpha t^2 e^{i\varphi}}{1-\alpha^2r^2} & \frac{r\beta^2}{1-\alpha^2r^2} & -\frac{\beta r\alpha t e^{-i\varphi_2}}{1-\alpha^2r^2} & -\frac{\beta t e^{-i\varphi_2}}{1-\alpha^2r^2} \\ -\frac{\beta t e^{i\varphi_1}}{1-\alpha^2r^2} & -\frac{\beta r\alpha t e^{i\varphi_2}}{1-\alpha^2r^2} & -\frac{\beta^2 r}{1-\alpha^2r^2} & \frac{\alpha t^2}{1-\alpha^2r^2} \\ -\frac{\beta r\alpha t e^{i\varphi_1}}{1-\alpha^2r^2} & -\frac{\beta t e^{i\varphi_2}}{1-\alpha^2r^2} & \frac{\alpha t^2}{1-\alpha^2r^2} & -\frac{\beta^2 r}{1-\alpha^2r^2} \end{pmatrix}, \tag{2}$$

where $\varphi \equiv \varphi_1 - \varphi_2$. As we show below, it is this phase difference between the modulation phases of the two pumps feeding the two parametric active devices (i.e., the JPCs), which induces the

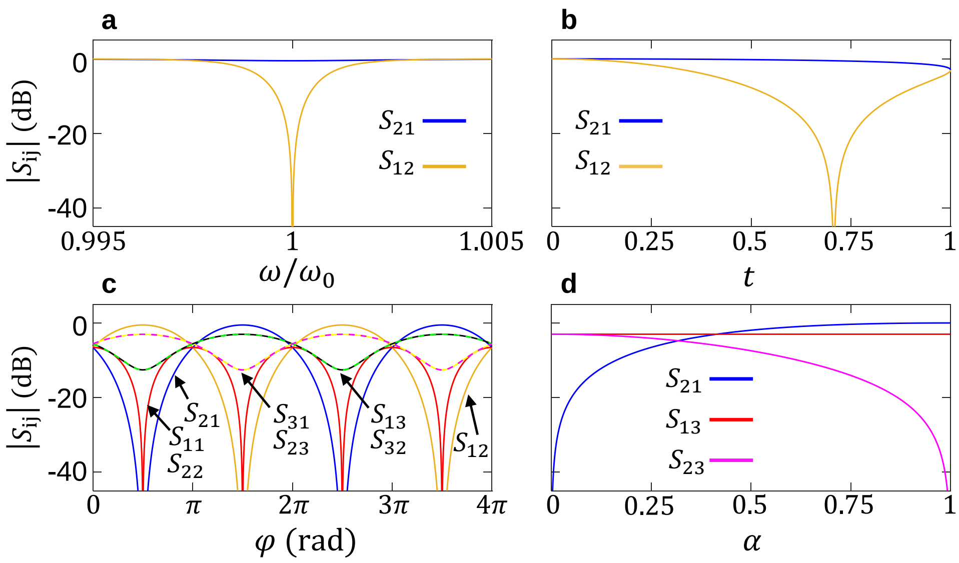

Supplementary Figure 2: **Theoretical results of the MPIJIS. a** Magnitude of the transmission parameters S_{21} (blue) S_{12} (orange) versus normalized angular frequency ω/ω_0, where ω_0 is the angular resonance frequency of resonator 'a'. **b** Magnitude of the transmission parameters S_{21} (blue) S_{12} (orange) on resonance versus the transmission parameter t of the balanced JPCs. In both cases **a** and **b**, the MPIJIS is biased in the forward direction ($\varphi = -\pi/2$). **c** Periodic response of the various scattering parameters on resonance versus the pump phase difference φ: $|S_{11}|^2$ and $|S_{22}|^2$ (red), $|S_{21}|^2$ (blue), $|S_{21}|^2$ (orange), $|S_{31}|^2$ (dashed yellow), $|S_{23}|^2$ (dashed magenta), $|S_{13}|^2$ (dashed black), and $|S_{32}|^2$ (dashed green). In the calculations **a** and **c**, the JPCs are operated at the 50:50 beam splitter working point. In the calculations **a**, **b**, and **c**, the effective coupler is assumed to be a hybrid with equal real coefficients $\alpha = \beta = 1/\sqrt{2}$. **d** $|S_{21}|^2$ (blue), $|S_{13}|^2$ (red), and $|S_{23}|^2$ (magenta) on resonance versus the effective coupler coefficient α, which is varied between 0 and 1. In this calculation, $\beta = \sqrt{1-\alpha^2}$ and t is calculated for each given α to yield a fixed isolation $|S_{12}|^2 = 0.01$ (-20 dB) on resonance.

nonreciprocal response of the MPIJIS [4–7]. The common coefficient $1/(1-\alpha^2 r^2)$ that appears in the scattering parameters of Eq. (2) represents the sum over all possible reflections that the internal signals can experience in the self-loop formed between the two b ports of the device. Unlike the directional amplification case [3, 8], where the reflection-gain amplitude needs to be bounded to ensure stability, in the case of frequency conversion, with no photon gain, the scattering parameters of Eq. (2) are stable for all values of $0 \leq r \leq 1$. In this simplified model, we assume that the phase acquired by signals at frequency f_2, propagating along the short transmission line between the two JPCs, is 2πk in each direction, where k is an integer. In our device, the electrical length of the short transmission line is designed to give a phase of about 2π at f_2.

It is straightforward to verify that the scattering matrix of Eq. (2) is unitary (energy preserving). For example, it satisfies the condition

$$|s_{1'1'}|^2 + |s_{1'2'}|^2 + |s_{1'3}|^2 + |s_{1'4}|^2 = 1. \tag{3}$$

Next, we derive the scattering matrix for the whole device, defined by ports 1, 2, 3, 4, which take into account the signal flow through the 90° hybrid,

$$[S] = \begin{pmatrix} S_{11} & S_{12} & S_{13} & S_{14} \\ S_{21} & S_{22} & S_{23} & S_{24} \\ S_{31} & S_{32} & S_{33} & S_{34} \\ S_{41} & S_{42} & S_{43} & S_{44} \end{pmatrix}, \tag{4}$$

whose matrix elements are given by,

$$S_{11} = \frac{1}{2}\left(s_{1'1'} - s_{2'2'} + is_{2'1'} + is_{1'2'}\right), \tag{5}$$

$$S_{12} = \frac{1}{2}\left(is_{1'1'} + is_{2'2'} + s_{1'2'} - s_{2'1'}\right), \tag{6}$$

$$S_{21} = \frac{1}{2}\left(is_{1'1'} + is_{2'2'} + s_{2'1'} - s_{1'2'}\right), \tag{7}$$

$$S_{22} = \frac{1}{2}\left(s_{2'2'} - s_{1'1'} + is_{2'1'} + is_{1'2'}\right), \tag{8}$$

$$\begin{aligned} S_{13} &= \tfrac{1}{\sqrt{2}}\left(is_{2'3} + s_{1'3}\right), \;\; S_{14} = \tfrac{1}{\sqrt{2}}\left(is_{2'4} + s_{1'4}\right), \\ S_{23} &= \tfrac{1}{\sqrt{2}}\left(s_{2'3} + is_{1'3}\right), \;\; S_{24} = \tfrac{1}{\sqrt{2}}\left(s_{2'4} + is_{1'4}\right), \end{aligned} \tag{9}$$

$$\begin{aligned} S_{31} &= \tfrac{1}{\sqrt{2}}\left(is_{32'} + s_{31'}\right), \;\; S_{32} = \tfrac{1}{\sqrt{2}}\left(s_{32'} + is_{31'}\right), \\ S_{41} &= \tfrac{1}{\sqrt{2}}\left(is_{42'} + s_{41'}\right), \;\; S_{42} = \tfrac{1}{\sqrt{2}}\left(s_{42'} + is_{41'}\right), \end{aligned} \tag{10}$$

$$\begin{aligned} S_{33} &= s_{33}, \;\; S_{34} = s_{34}, \\ S_{43} &= s_{43}, \;\; S_{44} = s_{44}. \end{aligned} \tag{11}$$

Note that $[S]$ is unitary because $[s]$ is unitary and the 90° hybrid is a unitary device. One prominent property seen from Eqs. (5)-(11), is the interferometric nature of the device, manifested in its scattering parameters, which represent the sum over all possible paths that the waves can propagate in it.

By substituting the scattering parameters of the inner device, listed in Eq. (2) into Eqs. (5)-(11), and by writing the resulting expressions in terms of the parameter t, we obtain the scattering parameters of the MPIJIS in an explicit form

$$S_{21} = \frac{i}{1+\frac{\alpha^2}{\beta^2}t^2}\left[\sqrt{1-t^2} - \frac{\alpha}{\beta^2}t^2\sin\varphi\right], \tag{12}$$

$$S_{12} = \frac{i}{1+\frac{\alpha^2}{\beta^2}t^2}\left[\sqrt{1-t^2} + \frac{\alpha}{\beta^2}t^2\sin\varphi\right], \tag{13}$$

$$S_{11} = S_{22} = -\frac{i\alpha}{\beta^2}\frac{t^2}{1+\frac{\alpha^2}{\beta^2}t^2}\cos\varphi, \tag{14}$$

$$S_{33} = S_{44} = -\frac{\sqrt{1-t^2}}{1+\frac{\alpha^2}{\beta^2}t^2}, \tag{15}$$

$$S_{34} = S_{43} = \frac{\alpha}{\beta^2}\frac{t^2}{1+\frac{\alpha^2}{\beta^2}t^2}, \tag{16}$$

$$S_{13} = -\frac{te^{-i\varphi_{\rm s}/2+i\pi/4}}{\sqrt{2}\beta\left(1+\frac{\alpha^2}{\beta^2}t^2\right)}\left[\sqrt{1-t^2}\alpha e^{i\frac{\varphi}{2}+i\frac{\pi}{4}} + e^{-i\frac{\varphi}{2}-i\frac{\pi}{4}}\right], \tag{17}$$

$$S_{14} = -\frac{te^{-i\varphi_{\rm s}/2+i\pi/4}}{\sqrt{2}\beta\left(1+\frac{\alpha^2}{\beta^2}t^2\right)}\left[e^{i\frac{\varphi}{2}+i\frac{\pi}{4}} + \sqrt{1-t^2}\alpha e^{-i\frac{\varphi}{2}-i\frac{\pi}{4}}\right], \tag{18}$$

$$S_{23} = -\frac{te^{-i\varphi_{\rm s}/2+i\pi/4}}{\sqrt{2}\beta\left(1+\frac{\alpha^2}{\beta^2}t^2\right)}\left[\sqrt{1-t^2}\alpha e^{i\frac{\varphi}{2}-i\frac{\pi}{4}} + e^{-i\frac{\varphi}{2}+i\frac{\pi}{4}}\right], \tag{19}$$

$$S_{24} = -\frac{te^{-i\varphi_{\rm s}/2+i\pi/4}}{\sqrt{2}\beta\left(1+\frac{\alpha^2}{\beta^2}t^2\right)}\left[e^{i\frac{\varphi}{2}-i\frac{\pi}{4}} + \sqrt{1-t^2}\alpha e^{-i\frac{\varphi}{2}+i\frac{\pi}{4}}\right], \tag{20}$$

$$S_{31} = -\frac{te^{i\varphi_s/2+i\pi/4}}{\sqrt{2}\beta\left(1+\frac{\alpha^2}{\beta^2}t^2\right)}\left[\sqrt{1-t^2}\alpha e^{-i\frac{\varphi}{2}+i\frac{\pi}{4}} + e^{i\frac{\varphi}{2}-i\frac{\pi}{4}}\right], \tag{21}$$

$$S_{32} = -\frac{te^{i\varphi_s/2+i\pi/4}}{\sqrt{2}\beta\left(1+\frac{\alpha^2}{\beta^2}t^2\right)}\left[\sqrt{1-t^2}\alpha e^{-i\frac{\varphi}{2}-i\frac{\pi}{4}} + e^{i\frac{\varphi}{2}+i\frac{\pi}{4}}\right], \tag{22}$$

$$S_{41} = -\frac{te^{i\varphi_s/2+i\pi/4}}{\sqrt{2}\beta\left(1+\frac{\alpha^2}{\beta^2}t^2\right)}\left[e^{-i\frac{\varphi}{2}+i\frac{\pi}{4}} + \sqrt{1-t^2}\alpha e^{i\frac{\varphi}{2}-i\frac{\pi}{4}}\right], \tag{23}$$

$$S_{42} = -\frac{te^{i\varphi_s/2+i\pi/4}}{\sqrt{2}\beta\left(1+\frac{\alpha^2}{\beta^2}t^2\right)}\left[e^{-i\frac{\varphi}{2}-i\frac{\pi}{4}} + \sqrt{1-t^2}\alpha e^{i\frac{\varphi}{2}+i\frac{\pi}{4}}\right], \tag{24}$$

where $\varphi_s \equiv \varphi_1 + \varphi_2$. In what follows, we examine a few special cases of interest and outline a few important properties of the device.

Without applied pump, i.e., $t = 0$, the MPIJIS scattering matrix reduces into

$$[S] = \begin{pmatrix} 0 & i & 0 & 0 \\ i & 0 & 0 & 0 \\ 0 & 0 & -1 & 0 \\ 0 & 0 & 0 & -1 \end{pmatrix}. \tag{25}$$

This result shows that when the device is OFF, the MPIJIS is transparent for propagating signals and effectively behaves as a lossless transmission line with an added reciprocal phase shift of $\pi/2$ for transmitted signals within the bandwidth of the 90° hybrid. In the special case, where the MPIJIS is ON and the phase difference between the pumps is $\varphi = -\pi/2$, the scattering matrix can be written in the form

$$[S] = \begin{pmatrix} 0 & i\Delta & -\sqrt{\frac{1-\Delta^2}{2}} & -\sqrt{\frac{1-\Delta^2}{2}} \\ i\Sigma & 0 & -i\sqrt{\frac{1-\Sigma^2}{2}} & i\sqrt{\frac{1-\Sigma^2}{2}} \\ -\sqrt{\frac{1-\Sigma^2}{2}} & -i\sqrt{\frac{1-\Delta^2}{2}} & -\frac{\Sigma+\Delta}{2} & \frac{\Sigma-\Delta}{2} \\ \sqrt{\frac{1-\Sigma^2}{2}} & -i\sqrt{\frac{1-\Delta^2}{2}} & \frac{\Sigma-\Delta}{2} & -\frac{\Sigma+\Delta}{2} \end{pmatrix}, \tag{26}$$

where $\Sigma \equiv g + h$, $\Delta \equiv g - h$, and

$$g = \frac{\sqrt{1-t^2}}{1+\frac{\alpha^2}{\beta^2}t^2}, \tag{27}$$

$$h = \frac{\alpha}{\beta^2}\frac{t^2}{1+\frac{\alpha^2}{\beta^2}t^2}. \tag{28}$$

In the derivation of Eq. (26), we assume, without loss of generality, that $\varphi_{\rm s} = \pi/2$.

When $\alpha = \beta = 1/\sqrt{2}$, $\varphi = -\pi/2$, and the JPCs are biased at the 50:50 beam splitter working point, the MPIJIS scattering matrix becomes

$$[S] = \begin{pmatrix} 0 & 0 & -\frac{1}{\sqrt{2}} & -\frac{1}{\sqrt{2}} \\ \frac{i2\sqrt{2}}{3} & 0 & -\frac{i}{3\sqrt{2}} & \frac{i}{3\sqrt{2}} \\ -\frac{1}{3\sqrt{2}} & -\frac{i}{\sqrt{2}} & -\frac{\sqrt{2}}{3} & \frac{\sqrt{2}}{3} \\ \frac{1}{3\sqrt{2}} & -\frac{i}{\sqrt{2}} & \frac{\sqrt{2}}{3} & -\frac{\sqrt{2}}{3} \end{pmatrix}. \tag{29}$$

A graphical representation of the scattering parameters of Eq. (29) is displayed in Supplementary Figure 1b. This result shows that when the MPIJIS is operated at this working point, it functions as an isolator with vanishing reflections $|S_{11}| = |S_{22}| = 0$, almost unity transmission in the forward direction $|S_{21}| = 2\sqrt{2}/3 \cong 0.943$ (which corresponds to an insertion loss of about 0.5 dB in the signal power), and total isolation $|S_{12}| = 0$. Furthermore, it shows that the cold loads on ports 3 and 4 play a similar role to internal ports of standard magnetic isolators. They dissipate the energy of back-propagating signals $|S_{32}| = |S_{42}| = 1/\sqrt{2}$ and emit noise (e.g., vacuum noise) towards the input $|S_{13}| = |S_{14}| = 1/\sqrt{2}$.

Another interesting case occurs when $t = \beta$ and $r = \alpha$. In this case, we get $|S_{21}| = 2\alpha/(1+\alpha^2)$. This result shows that $2\sqrt{2}/3$ is not a fundamental upper bound on $|S_{21}|$. In fact, for a highly uneven effective coupler with $\alpha \approx 1$, $\beta \approx 0$ and JPCs operated with little conversion $r \to 1$, $t \to 0$, we obtain $|S_{21}| \to 1$.

To generalize the on-resonance-derived scattering parameters listed in Eqs. (12)-(24) for signals within the device bandwidth, we substitute [2]

$$t[\omega_1] = \frac{2\rho}{\chi_{\rm a}^{-1}\chi_{\rm b}^{-1} + \rho^2}, \tag{30}$$

where $\chi' s$ are the bare response functions of modes a and b (whose inverses depend linearly on f_1 and f_2):

$$\chi_{\rm a}^{-1}[\omega_1] = 1 - 2i\frac{\omega_1 - \omega_{\rm a}}{\kappa_{\rm a}},$$
$$\chi_{\rm b}^{-1}[\omega_2] = 1 - 2i\frac{\omega_2 - \omega_{\rm b}}{\kappa_{\rm b}}. \tag{31}$$

Since the applied pump frequency satisfies the relations $\omega_{\rm p} = \omega_{\rm b} - \omega_{\rm a} = \omega_2 - \omega_1$, $\chi_{\rm b}^{-1}$ of Eq. (31) can be rewritten as $\chi_{\rm b}^{-1}[\omega_1] = 1 - 2i(\omega_1 - \omega_{\rm a})/\kappa_{\rm b}$. Obviously, this generalization holds under the assumption that the bandwidth of the 90° hybrid is much larger than the dynamical bandwidths of the JPCs, which is generally the case because transmission-line-based hybrids exhibit bandwidths of a few hundreds of megahertz [9].

In Supplementary Figure 2, we present several key theoretical results of the MPIJIS. Supplementary Figure 2a shows the transmission parameters magnitude $|S_{21}|^2$ (blue) and $|S_{12}|^2$ (orange) of the MPIJIS, operated in the forward direction, as a function of normalized frequency, where $\omega_0 = \omega_a$. As expected, $|S_{12}|^2$ exhibits a large dip in vicinity of the resonance while $|S_{21}|^2$ remains very close to unity. Supplementary Figure 2b displays $|S_{21}|^2$ and $|S_{12}|^2$ of a forward-operated MPIJIS on resonance as a function of the JPC transmission parameter t. In Supplementary Figure 2c, we show the dependence of a rather comprehensive set of the scattering parameters on the pump phase difference φ, namely, $|S_{11}|^2$ (red), $|S_{21}|^2$ (blue), $|S_{12}|^2$ (orange), $|S_{31}|^2$ (dashed yellow), $|S_{23}|^2$ (dashed magenta), $|S_{13}|^2$ (dashed black), and $|S_{32}|^2$ (dashed green). Note that some of the absent scattering parameters are equal in magnitude to ones that are present, i.e., $|S_{22}|^2 = |S_{11}|^2$, $|S_{41}|^2 = |S_{31}|^2$, $|S_{24}|^2 = |S_{23}|^2$, $|S_{14}|^2 = |S_{13}|^2$, $|S_{42}|^2 = |S_{32}|^2$. Similar to the calculation of Supplementary Figure 2a,b the different scattering parameters in Supplementary Figure 2c are calculated using Eqs. (12), (13), (14), (17), (19), (21), (22) for the case of a symmetrical effective coupler. Also, in Supplementary Figures 2a,c, the JPCs are operated at the 50:50 beam splitter point. As expected for $\varphi = -\pi/2 + 2\pi$k, where k is an integer, $|S_{21}|^2$ (forward transmission) assumes a maximum, while $|S_{11}|^2$ (reflection), $|S_{12}|^2$ (backward transmission) assume a minimum. Similarly, $|S_{13}|^2$ (transmission from the load to the input) and $|S_{32}|^2$ (transmission from the output to the load) assume a maximum, while $|S_{31}|^2$ (transmission from the input to the load) and $|S_{23}|^2$ (transmission from the load to the output) assume a minimum. The responses are reversed for $\varphi = \pi/2 + 2\pi$k. Finally, Supplementary Figure 2d displays a representative set of the scattering parameters of the MPIJIS on resonance versus the transmission amplitude between modes b of the JPCs, i.e., α. In this calculation, the MPIJIS is operated in the forward direction and the transmission parameter of the JPCs t is adjusted for each chosen α ($t \cong \beta$) to yield a fixed attenuation of 20 dB in the backward direction $|S_{12}|^2$. As seen in the figure, $|S_{21}|^2$ asymptotically approaches 1 in the limit

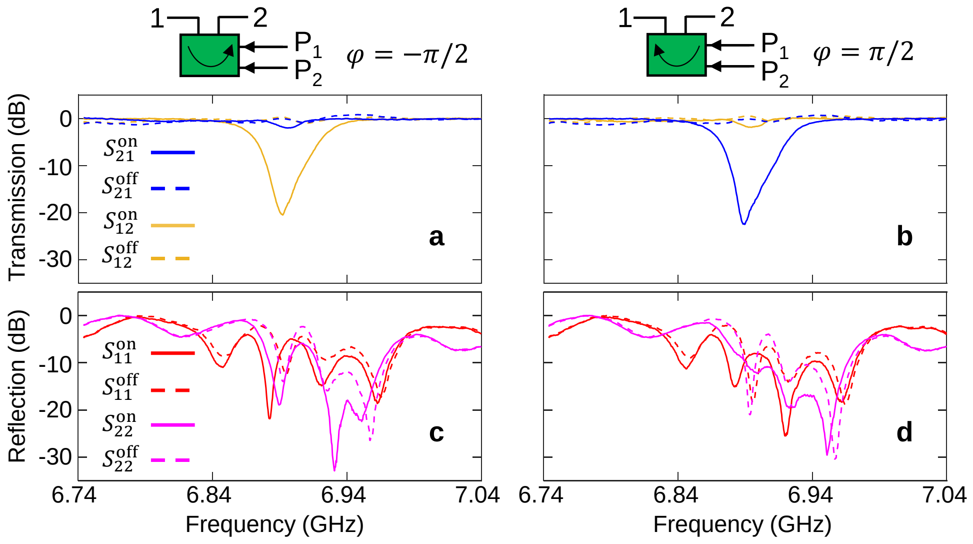

Supplementary Figure 3: Measured MPIJIS scattering parameters versus frequency. **a** and **b** show the transmission parameters $|S_{21}|^2$ (solid blue) and $|S_{12}|^2$ (solid orange) measured for the MPIJIS biased in the forward ($\varphi = -\pi/2$) and backward ($\varphi = \pi/2$) directions, respectively. **c** and **d** show the corresponding reflection parameters $|S_{11}|^2$ (solid red) and $|S_{22}|^2$ (solid magenta). The dashed curves in **a-d** correspond to the MPIJIS being in the OFF state. The applied pump frequency in this measurement is 2.8 GHz.

$\alpha \to 1$. Moreover, while $|S_{13}|^2$ (transmission from the load to the input), represented by the red line, remains close to -3 dB, $|S_{23}|^2$ (transmission from the load to the output), represented by the magenta line, approaches 0 in the limit $\alpha \to 1$.

Supplementary Note 2: Characterization measurements of the MPIJIS

In Supplementary Figure 3, we exhibit scattering parameters measurement of the MPIJIS versus frequency taken while operated in the forward direction $\varphi = -\pi/2$ (Supplementary Figure 3a, 3c) and the backward direction $\varphi = \pi/2$ (Supplementary Figures 3b, 3d). In this measurement, taken in a separate cooldown, the MPIJIS is characterized directly without the qubit setup. The solid (dashed) curves represent measurements taken with the MPIJIS ON (OFF). The blue, orange, red, and magenta curves represent $|S_{21}|^2$, $|S_{12}|^2$, $|S_{11}|^2$, and $|S_{22}|^2$, respectively. When the MPIJIS is OFF, both transmission parameters are close to unity (≈ 0 dB) and the reflection parameters are suppressed (below -10 dB), which is consistent with the MPIJIS being transparent for transmitted signals between ports 1 and 2 in the OFF state. When the MPIJIS is operated in the forward

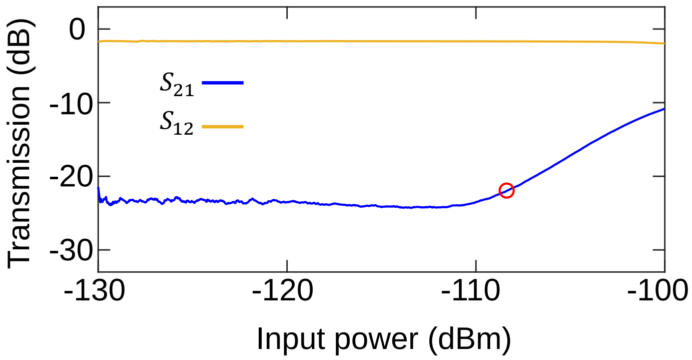

Supplementary Figure 4: Maximum input power measurement of the MPIJIS. In this measurement, the MPIJIS is biased in the backward direction on resonance as shown in Supplementary Figure 3b. The red circle indicates the saturation power at which the isolation magnitude of the device represented by the blue line ($|S_{21}|^2$) decreases by 1 dB compared to the low-power value. In comparison, $|S_{12}|^2$ represented by the orange line remains flat even beyond the saturation power.

direction (Supplementary Figure 3a), $|S_{21}|^2$ remains relatively close to unity ~ -1.8 dB, while $|S_{12}|^2$ exhibits a dip of -20.3 dB on resonance and a dynamical bandwidth of 11 MHz. When the MPIJIS is operated in the backward direction, the roles played by the transmission parameters S_{21} and S_{12} are reversed as seen in Supplementary Figure 3b. Moreover, the reflection parameters $|S_{11}|^2$, and $|S_{22}|^2$ are suppressed further on resonance, as expected, when the MPIJIS is ON, as seen in Supplementary Figures 3c and 3d.

Furthermore, we measure the maximum input power which the MPIJIS can handle on resonance, at a given isolation (e.g., 20 dB), above which it saturates. The measurement result taken for the MPIJIS operated in the backward direction (same working point as Supplementary Figure 3b) is shown in Supplementary Figure 4. The plot depicts the transmission parameters $|S_{21}|^2$ (blue) and $|S_{12}|^2$ (orange) as a function of input power. As seen in the figure, $|S_{12}|^2$ is almost flat around -1.5 dB up to -100 dBm, whereas $|S_{21}|^2$ is almost flat around -22 dB in the range between -130 to -110 dBm but it gradually degrades beyond -110 dBm. One figure of merit which we apply here to quantify the saturation power of the isolator is the 1 dB compression point (denoted as $P_{-1\mathrm{dB}}$), which is commonly used to characterize amplifiers. In the amplifier case, the 1 dB compression point usually corresponds to the input power for which the low-input-power-gain of the amplifier degrades by -1 dB. Analogously, the 1 dB compression point in our Josephson isolator case corresponds to the input power for which the isolation degrades by $+1$ dB (denoted as $P_{+1\mathrm{dB}}$).

In our device, we find $P_{+1\mathrm{dB}} = -108$ dBm, which is indicated by a red circle in Supplementary Figure 4. It is worth noting that this figure is significantly larger than $P_{-1\mathrm{dB}}$ of microstrip-based JPCs, when operated in the amplification mode, which is on the order of -130 dBm [2, 10]. This is because in the isolator case the JPCs are operated in the frequency conversion mode (without photon gain) [11].

It is worth pointing out that although the MPIJIS employs two JPCs, two external coils, and two input pumps, the device can be quite stable over time provided it is positioned at the bottom of a deep cryoperm magnetic shield can and the microwave generators feeding the pumps are phase-locked to the 10 MHz reference oscillator of a rubidium atomic clock. By using two phase-locked generators, as done in this work, the set working point can stay stable over a few hours at most. To further extend the device stability over days, one can feed the two pumps using the same generator by splitting its output into two arms and incorporating a tunable attenuator and phase shifter into one arm, as was demonstrated in the directional amplifier experiment [8].

Supplementary Note 3: Added noise

To calculate the added noise by the MPIJIS operated in the forward direction, we compare the signal-to-noise ratio at the output S_2/N_2 to the signal-to-noise ratio at the input S_1/N_1, where S_i and N_i represent the number of signal and noise-equivalent photons per mode per unit time per unit bandwidth at port 'i', respectively [2]. Using the full scattering matrix of the MPIJIS (Eq. (4)), we write $S_2 = |S_{21}|^2 S_1$, $N_2 = |S_{21}|^2 N_1 + |S_{23}|^2 N_3 + |S_{24}|^2 N_4 = N_1$, where the last equality holds because the scattering matrix is unitary and $S_{22} = 0$. We also assume here that the dominant noise entering the system is vacuum noise. Using these relations, we obtain for the noise factor $\mathrm{NF} = (\mathrm{S_2/N_2})/(\mathrm{S_1/N_1}) = |\mathrm{S_{21}}|^2$. Alternatively, NF can be expressed in terms of the number of noise-equivalent photons added by the MPIJIS to the input n_add, where $S_2 = |S_{21}|^2 S_1$ and $N_2 = |S_{21}|^2 (N_1 + n_\mathrm{add})$. In this representation, $\mathrm{NF} = \mathrm{N_1/(N_1 + n_{add})}$. Solving for n_add, gives $n_\mathrm{add} = (1 - |S_{21}|^2)/2|S_{21}|^2$, where we substituted the vacuum-noise contribution $N_1 = 1/2$. Based on this result, we estimate the added noise by the MPIJIS in the qubit measurement of Fig.4b to be $n_\mathrm{add} \simeq 0.1$, obtained at $|S_{21}|^2 = 0.85$ (corresponding to about 0.7 dB dip on resonance observed in Fig. 4b in the main text).

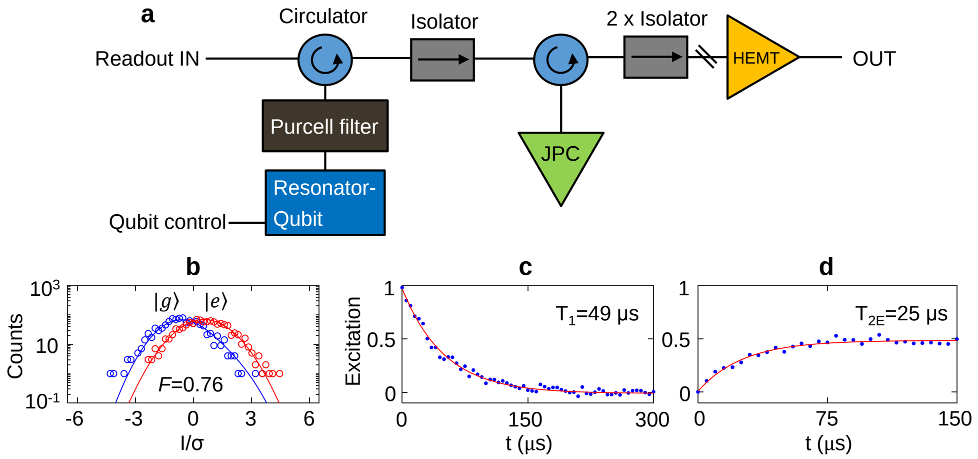

Supplementary Figure 5: Qubit characterization with a conventional readout setup. a Block-circuit diagram of a conventional rapid, high-fidelity qubit readout setup, which utilizes magnetic-based circulators and isolators. **b**, **c**, and **d** exhibit qubit measurements, i.e., qubit readout fidelity, T_1, and $T_{2\mathrm{E}}$, respectively, taken in a separate cooldown using the same qubit-resonator device and measurement parameters as Fig. 4 in the main text. The JPC is turned off in these measurements.

Supplementary Note 4: Comparison to conventional high fidelity qubit setup

In Supplementary Figure 5a, we exhibit a block-circuit diagram of a conventional rapid, high-fidelity qubit readout setup, which employs magnetic-based circulators and isolators. In Supplementary Figure 5b-d, we exhibit qubit measurements corresponding to qubit readout fidelity, T_1, and $T_{2\mathrm{E}}$, respectively, taken in a separate cooldown with the conventional setup displayed in Supplementary Figure 5a. All three measurements shown in Supplementary Figures 5b-d are taken with the JPC turned off using the same qubit-resonator-Purcell filter system and measurement parameters of Fig. 4 in the main text. As seen in Supplementary Figures 5b-d, the measured readout fidelity $F = 0.75$, qubit lifetime $T_1 = 49$ μs, and coherence time $T_{2\mathrm{E}} = 25$ μs are comparable with those measured for configuration **a** of Fig. 4 in the main text, which uses the superconducting directional coupler and MPIJIS instead of the qubit-circulator and intermediate isolator shown in Supplementary Figure 5a. Note that although $T_1 = 49$ μs measured with the conventional setup of Supplementary Figure 5a is slightly higher than $T_1 = 34$ μs measured with the setup of Fig. 3, they fall within the variation range observed for different cooldowns. It is worth noting that turning on the JPC in this conventional setup, which has three isolating stages between the JPC and the qubit-resonator, does not degrade T_1 or $T_{2\mathrm{E}}$, shown in Supplementary Figures 5c,d. It only

enhances the measurement fidelity shown in Supplementary Figure 5b to about $F = 0.98$.

Supplementary Note 5: Qubit decoherence due to generated heat in the MPIJIS pump lines

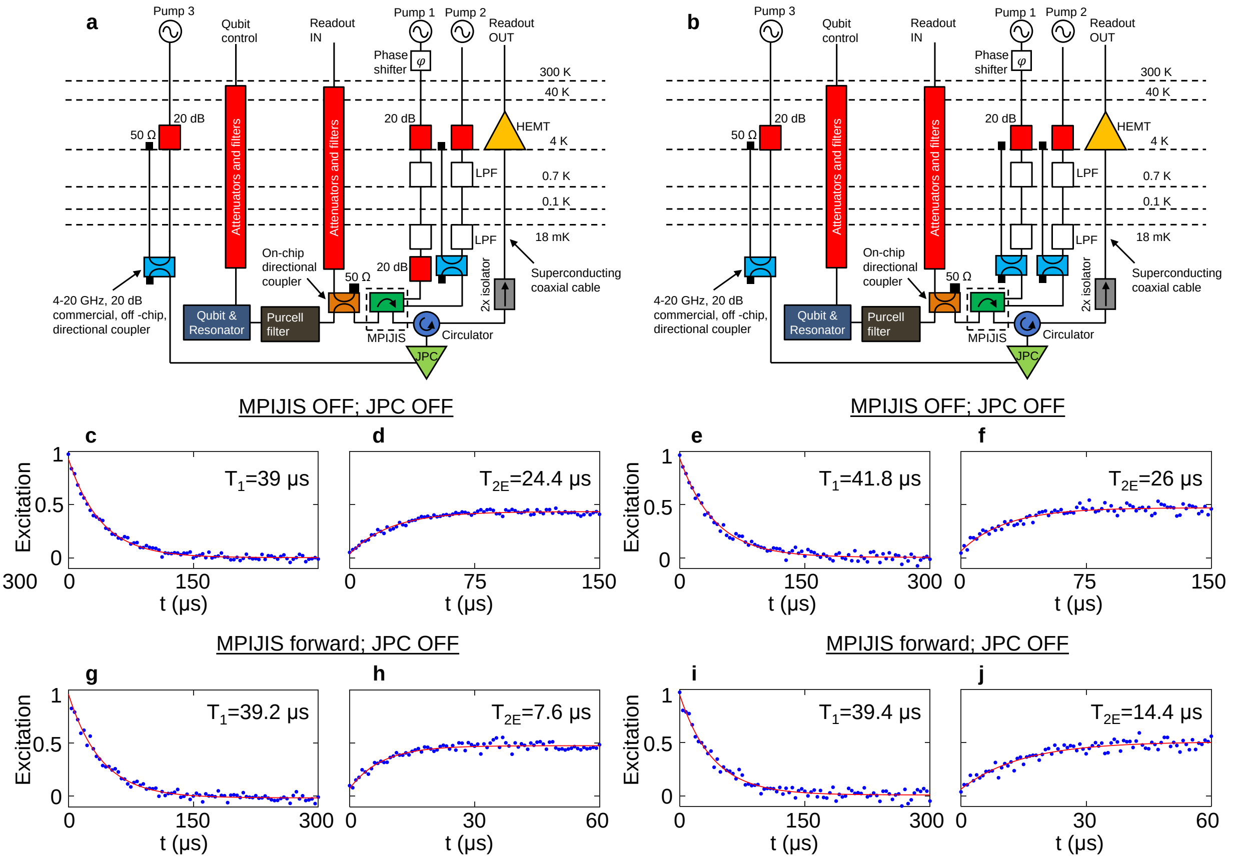

Supplementary Figure 6: Qubit decoherence due to generated heat in the MPIJIS pump lines. **a** Preliminary experimental setup used for measuring the qubit with the MPIJIS and JPC. One of the pump lines (i.e., pump 1) feeding the MPIJIS includes a 20 dB resistive attenuator at the base-temperature stage. The other pump line (i.e., pump 2) includes a 20 dB directional coupler at the bottom stage, which attenuates the pump by routing most of its power to a higher-temperature stage, where it gets dissipated in a 50 Ohm termination. **b** The experimental setup used for taking the main results of the paper shown in Fig. 4 and Table I. Here, the 20 dB resistive attenuator at the base-temperature stage used on pump 1 is replaced by a 20 dB directional coupler similar to pump 2. **c**, **d** exhibit T_1, and $T_{2\mathrm{E}}$ measurements taken using setup **a** while the MPIJIS and JPC are OFF. **e**, **f** exhibit similar measurements taken using setup **b**. **g**, **h** exhibit T_1, and $T_{2\mathrm{E}}$ measurements taken using setup **a**, while the MPIJIS is ON and biased in the forward direction. **i**, **j** exhibit similar measurements taken using setup **b**.

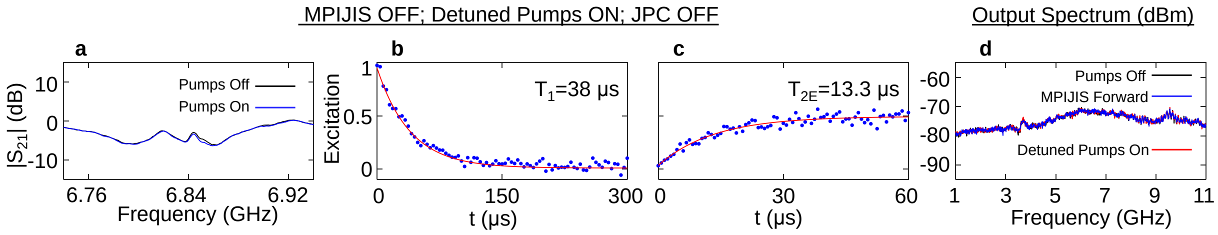

Supplementary Figure 7: Detuned-pumps experiment and spectrum measurement. **a** transmission parameter measurements of the MPIJIS taken with the setup shown in Supplementary Figure 6**b**, corresponding to the MPIJIS being OFF (black curve) and pumped at a detuned frequency (blue curve). **b**, **c** exhibit T_1, and $T_{2\mathrm{E}}$ measurements taken while the MPIJIS is driven by detuned pumps. **d** Broadband spectrum measurements of the MPIJIS output port, taken with a spectrum analyzer using the same cryogenic setup. The black, blue, and red curves correspond to the MPIJIS being OFF, biased in the forward direction, and driven by detuned pumps, respectively.

Preliminary qubit-MPIJIS measurements taken using the wiring scheme depicted in Supplementary Figure 6a, yielded significant degradation of $T_{2\mathrm{E}}$ of the qubit when the MPIJIS is ON (i.e, 7.6 μs shown in Supplementary Figure 6h) versus OFF (i.e., 24.4 μs shown in Supplementary Figure 6d), while maintaining T_1 of about 39 μs as demonstrated in Supplementary Figure 6c and Supplementary Figure 6g. As seen in Supplementary Figure 6a, featuring the main components, the wiring setup for the experiment consists of two attenuated and filtered input lines for controlling and measuring the qubit, one for outputting the measured quantum signal, which contains wideband isolators and a low-noise semiconductor-based amplifier (i.e., HEMT), and three input lines for feeding the pump drives to the JPC (one line) and the MPIJIS (two lines). It is worth noting that the pump input lines include 20 dB of resistive-based attenuation at the 4 K stage and another 20 dB at the base-temperature stage, where the qubit and MPIJIS are mounted, which take the form of either a resistive-based attenuator installed on pump 1 or a dispersive-based attenuator, i.e., commercial broadband directional coupler, installed on pumps 2 and 3. The main advantage of using the dispersive-based attenuator, i.e., the directional coupler, at the base-temperature stage is that, unlike the resistive-based attenuator, which attenuates the pump by dissipating a portion of its power, the directional coupler attenuates the pump by routing a portion of its power to a separate port, which, in turn, can be carried by via a coaxial line and dissipated at a 50 Ohm termination located at a higher-temperature stage with a larger cooling power, i.e., 4 K stage.

Following these results, we have run several experiments (three of which are outlined here)

to uncover the main cause for the $T_{2\mathrm{E}}$ degradation due to the operation of the MPIJIS. In one experiment, we substituted the 20 dB resistive-based attenuator at the base-temperature stage on pump 1 with a commercial, broadband 20 dB directional coupler and a pump return line terminated at the 4 K stage as depicted in Supplementary Figure 6b. By implementing this sole change in the setup, we saw a significant enhancement of $T_{2\mathrm{E}}$ by about a factor 2 when the MPIJIS is ON (i.e., 14.4 μs shown in Supplementary Figure 6j) compared to the original setup and cooldown (i.e., 7.6 μs shown in Supplementary Figure 6h). Moreover, as seen in Supplementary Figure 6e and Supplementary Figure 6f, this change in the setup did not alter the baseline values for T_1 and $T_{2\mathrm{E}}$ (when both the JPC and MPIJIS are OFF) compared to the original setup, neither did it affect T_1 when the MPIJIS is ON (as shown in Supplementary Figure 6i) versus OFF. Such $T_{2\mathrm{E}}$ enhancement observed in this setup suggests that excess heat generated in the pump lines (when the MPIJIS) is ON, increases the thermal photon population in the readout resonator, which, in turn, increases the qubit dephasing, thus degrading its $T_{2\mathrm{E}}$. This pump-generated heating hypothesis is further supported by the observed increase of $0.2 - 3$ mK in the base-stage temperature when the MPIJIS pumps are ON versus OFF (the observed increase is smaller in setup **b** than **a**). It is worth noting that the main results of the paper, shown in Fig. 4 in the main text, are obtained using the setup shown in Supplementary Figure 6b.

Furthermore, to rule out the possibility that the decrease in $T_{2\mathrm{E}}$ is caused by the MPIJIS operation rather than due to heat generated in the pump lines, we applied frequency-detuned pump drives to the MPIJIS, whose frequency is 380 MHz lower than that of Supplementary Figure 6i and Supplementary Figure 6j, while maintaining the same source powers. As seen in Supplementary Figure 7a, which plots the transmission parameter through the MPIJIS versus frequency, applying the detuned pumps to the MPIJIS does not change its response and it does not turn on the device. This OFF state of the MPIJIS, was maintained when the phase difference between the two pumps is varied and when either pump is applied individually. As expected, applying these comparable pump-powers to the device without turning on the MPIJIS yielded $T_1 = 38$ μs (shown in Supplementary Figure 7b) and $T_{2\mathrm{E}} = 13.3$ μs (shown in Fig. 7c), which are similar to the values observed in Supplementary Figure 6i and Supplementary Figure 6j, respectively.

Finally, to verify that the MPIJIS does not generate spurious harmonics, noise, or leaks out pump photons or internal mode photons (i.e., mode b), which could potentially have a negative effect on $T_{2\mathrm{E}}$, we present in Supplementary Figure 7d a broadband spectrum measured at the output of the MPIJIS port using the wiring setup of Supplementary Figure 6b. The black and blue curves are data taken using a spectrum analyzer while the MPIJIS is in the OFF and ON states,

respectively. In the ON state, the MPIJIS is operated at the same working point as in Supplementary Figures. 6i,j. The red curve represents the output spectrum measured for the detuned pumps case corresponding to working point of Supplementary Figures 7a-c. As seen in the figure, the three measured curves are indistinguishable, which implies that the MPIJIS does not generate any observable spurious harmonics or noise when it is ON.

We also estimate, based on the generators output and the total attenuation of the lines, that the microwave power dissipated at the mixing chamber by the MPIJIS pump drives falls in the range $10^{-5} - 10^{-7}$ W for the configuration with the resistive attenuator, shown in Supplementary Figure 6a, and $10^{-7} - 10^{-9}$ W for the configuration with the directional couplers, shown in Supplementary Figure 6b.

Additional steps that can be taken to further reduce the dissipated power at the base-temperature stage and consequently eliminate the degradation of the qubit coherence, when the MPIJIS is ON, include: 1) replacing the normal-metal directional couplers on the pump lines with superconducting versions, 2) routing and dissipating the reflected pump power off the MPIJIS at a higher-temperature stage, e.g., the 100 mK stage, and 3) redesigning the on-chip pump feedline, illustrated in Fig. 2c in the main text, to allow the JPC to operate in frequency conversion mode at lower pump powers.

---

[1] Pozar, D. M. Microwave Engineering, 3rd edition, (Wiley, Hoboken, NJ, 2005).

[2] Abdo, B., Kamal, A. and Devoret, M. H. Nondegenerate three-wave mixing with the Josephson ring modulator. *Phys. Rev. B* **87**, 014508 (2013).

[3] Abdo, B., Sliwa, K., Shankar, S., Hatridge, M., Frunzio, L., Schoelkopf, R. and Devoret, M. Josephson Directional Amplifier for Quantum Measurement of Superconducting Circuits. *Phys. Rev. Lett.* **112**, 167701 (2014).

[4] Kamal, A., Clarke, J. and Devoret, M. Noiseless non-reciprocity in a parametric active device. *Nat. Phys.* **7**, 311 (2011).

[5] Fang, K., Yu, Z. and Fan, S. Photonic Aharonov-Bohm Effect Based on Dynamic Modulation. *Phys. Rev. Lett.* **108**, 153901 (2012).

[6] Fang, K., Yu, Z. and Fan, S. Experimental demonstration of a photonic Aharonov-Bohm effect at radio frequencies. *Phys. Rev. B* **87**, 060301(R) (2013).

[7] Abdo, B., Brink, M., Chow, J. M., Gyrator Operation Using Josephson Mixers. *Phys. Rev. Applied* **8**, 034009 (2017).

[8] Abdo, B., Bronn, N., Jinka, O., Olivadese, S., Brink, M., Chow, J. M. Multi-path interferometric

Josephson directional amplifier for qubit readout. *Quantum Sci. Technol.* **3**, 024003 (2018).

[9] Ku, H. S., Mallet, F., Vale, L. R., Irwin, K. D., Russek, S. E., Hilton, G. C. and Lehnert, K. W. Design and Testing of Superconducting Microwave Passive Components for Quantum Information Processing. *IEEE Trans. on Appl. Supercond.* **21**, 452 (2010).

[10] Abdo, B., Chavez-Garcia, J. M., Brink, M., Keefe, G. and Chow, J. M. Time-multiplexed amplification in a hybrid-less and coil-less Josephson parametric converter. *Appl. Phys. Lett.* **110**, 082601 (2017).

[11] Abdo, B. , Sliwa, K., Schackert, F., Bergeal, N., Hatridge, M., Frunzio, L., Stone, A. D. and Devoret, M. Full Coherent Frequency Conversion between Two Propagating Microwave Modes. *Phys. Rev. Lett.* **110**, 173902 (2013).
